# Targeting protein biotinylation enhances tuberculosis chemotherapy

**DOI:** 10.1126/scitranslmed.aal1803

**Published:** 2018-04-25

**Authors:** Divya Tiwari, Sae Woong Park, Maram M. Essawy, Surendra Dawadi, Alan Mason, Madhumitha Nandakumar, Matthew Zimmerman, Marizel Mina, Hsin Pin Ho, Curtis A. Engelhart, Thomas Ioerger, James C. Sacchettini, Kyu Rhee, Sabine Ehrt, Courtney C. Aldrich, Véronique Dartois, Dirk Schnappinger

**Affiliations:** 1 Department of Microbiology and Immunology, Weill Cornell Medical College, New York, NY 10021, USA; 2 Department of Medicinal Chemistry, University of Minnesota, 308 Harvard Street Southeast, 8-174 WDH, Minneapolis, MN 55455, USA; 3 Public Health Research Institute, New Jersey Medical School, Rutgers, State University of New Jersey, Newark, NJ 07103, USA; 4 Weill Department of Medicine, Weill Cornell Medical College, New York, NY 10021, USA; 5 Department of Computer Science and Engineering, Texas A&M University, College Station, TX 77843, USA; 6 Department of Biochemistry and Biophysics, Texas A&M University, College Station, TX 77843, USA; 7 Department of Medicine, New Jersey Medical School, Rutgers, State University of New Jersey, Newark, NJ 07103, USA

## Abstract

Successful drug treatment for tuberculosis (TB) depends on the unique contributions of its component drugs. Drug resistance poses a threat to the efficacy of individual drugs and the regimens to which they contribute. Biologically and chemically validated targets capable of replacing individual components of current TB chemotherapy are a major unmet need in TB drug development. We demonstrate that chemical inhibition of the bacterial biotin protein ligase (BPL) with the inhibitor Bio-AMS (5′-[*N*-(d-biotinoyl)sulfamoyl]amino-5′-deoxyadenosine) killed *Mycobacterium tuberculosis* (*Mtb*), the bacterial pathogen causing TB. We also show that genetic silencing of BPL eliminated the pathogen efficiently from mice during acute and chronic infection with *Mtb*. Partial chemical inactivation of BPL increased the potency of two first-line drugs, rifampicin and ethambutol, and genetic interference with protein biotinylation accelerated clearance of *Mtb* from mouse lungs and spleens by rifampicin. These studies validate BPL as a potential drug target that could serve as an alternate frontline target in the development of new drugs against *Mtb*.

## INTRODUCTION

Tuberculosis (TB) is re-emerging as an incurable infection due to drug resistance. In 2013, about 480,000 people developed multidrug-resistant tuberculosis (MDR-TB) (*1*). The discovery of an effective vaccine that prevents TB in adults remains an important goal but has been elusive (*2*). Consequently, our ability to control TB depends primarily on the development of more efficient chemotherapies for drug-sensitive and drug-resistant TB.

The cell envelope of mycobacteria is a selective permeability barrier containing several unique lipids that confer drug resistance on *Mycobacterium tuberculosis* (*Mtb*), the bacterial pathogen that causes TB, and protect it from the host immune system (*3**–**7*). The importance of mycobacterial lipid metabolism is underscored by the finding that more than 250 genes are involved in lipid metabolism in *Mtb* as opposed to only 50 in *Escherichia col****i***. The structurally diverse mycobacterial lipids are all derived from simple malonyl coenzyme A (CoA) building blocks, which are, in turn, made by acyl-CoA carboxylases (ACCs). *Mtb* encodes three multimeric ACCs assembled from at least 10 different subunits (AccA1 to AccA3, AccD1 to AccD6, and AccE5), which together provide the malonyl-CoA, (methyl)malonyl CoA, and (long-chain alkyl)malonyl CoA building blocks required for synthesis of linear fatty acids, methyl-branched lipids, and mycolates, respectively (*8**–**11*). Each ACC must be post translationally modified with the cofactor biotin (also known as vitamin H or vitamin B7) to become active. Blocking de novo biotin biosynthesis or biotin-ACC ligation thus has the potential to inhibit all lipid biosynthesis in mycobacteria.

**Fig. 3:**
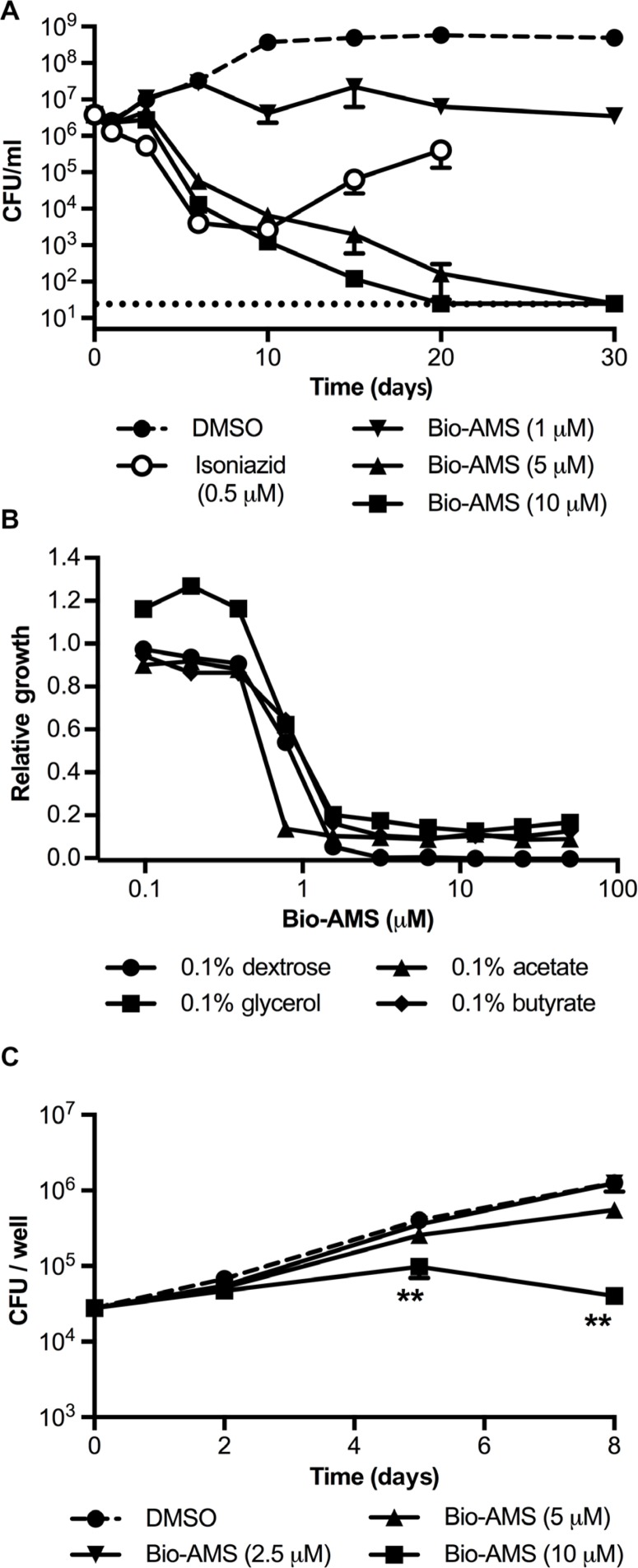
**Activity of Bio-AMS in a hollow fiber culture system.** (**A**) PK profile of Bio-AMS in a hollow fiber culture system. Bio-AMS concentrations were measured in samples taken from the central reservoir and the extracapillary space of the device. The dotted line represents the MIC_90_ of Bio-AMS for the *Mtb* H37Ra strain. (**B**) Impact of Bio-AMS on viability of the *Mtb* H37Ra strain in a hollow fiber culture system. The dotted line indicates the lower limit of detection.

We previously reported the design and characterization of potent inhibitors of biotin protein ligase (BPL), the enzyme responsible for covalently ligating biotin onto the ACCs (*12*–*19*). This led to 5′-[*N*-(d-biotinoyl)sulfamoyl]amino-5′-deoxyadenosine (Bio-AMS), a BPL inhibitor with potent on-target whole-cell activity against drug-sensitive and drug-resistant *Mtb* (*17*). Nevertheless, several important questions remained concerning its mechanism of action, mechanism of resistance in *Mtb*, pharmacokinetic (PK) properties, synergy with other antitubercular drugs, and, importantly, consequences of BPL inhibition in vivo.

Here, we first characterized the consequences of chemically inactivating BPL for *Mtb* viability under different physiological conditions. Next, we determined the frequency and mechanism of resistance to BPL inactivation by Bio-AMS and then used PK studies to characterize Bio-AMS metabolism in mice. Incomplete inhibition of BPL due to fluctuating concentrations of Bio-AMS prevented growth of *Mtb* in a hollow fiber culture system. Furthermore, near-complete genetic inactivation of BPL killed *Mtb* during acute and chronic infection of mice. Finally, we established that partial genetic interference with protein biotinylation was sufficient to increase sensitivity of *Mtb* to killing by rifampicin during infection of mice.

## RESULTS

### Impact of chemical BPL inactivation on *Mtb* viability in vitro

**Fig. 1:**
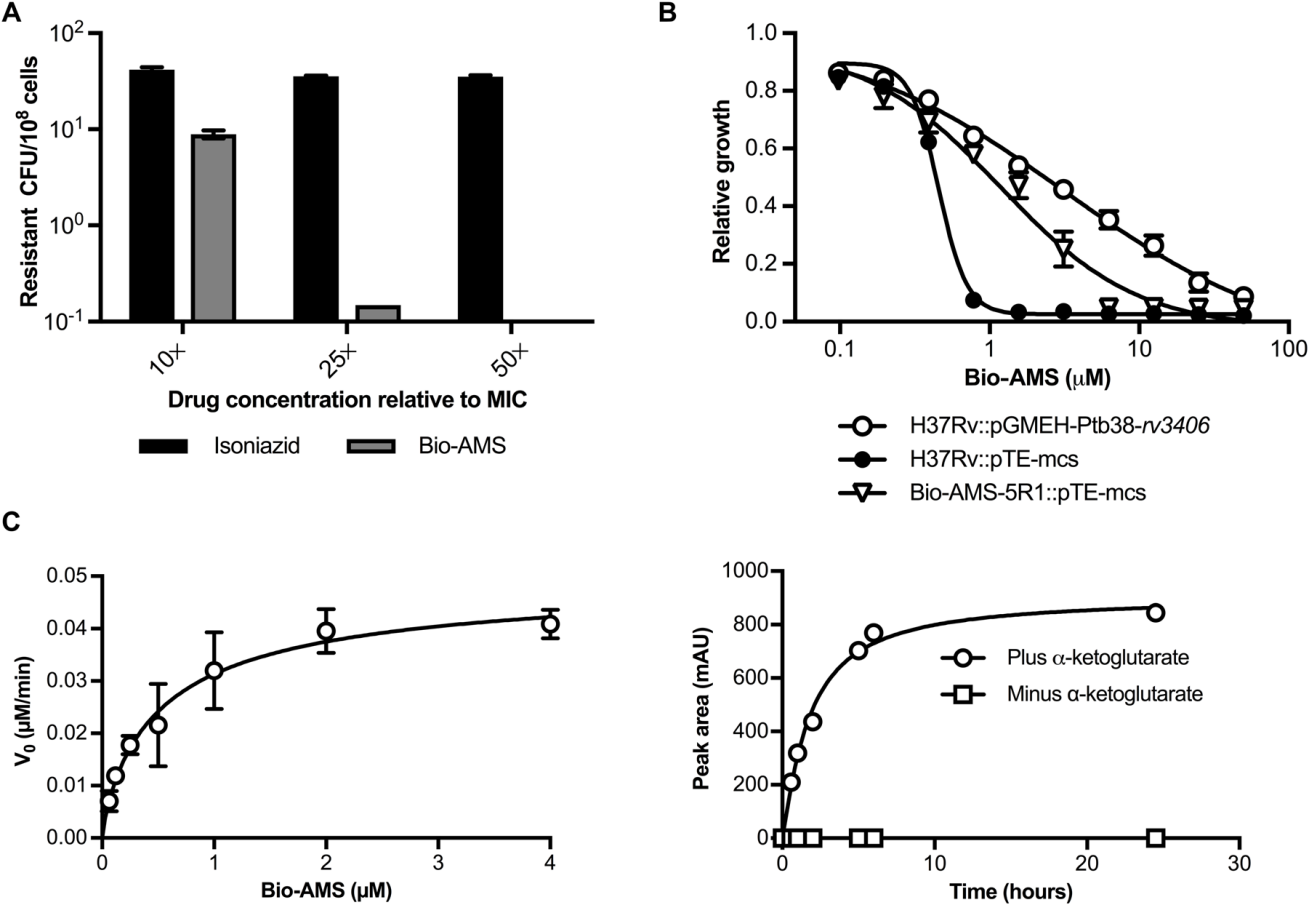
**Chemical inactivation of BPL kills *Mtb* and prevents growth in mouse macrophages.** (**A**) Impact of Bio-AMS on viability of *Mtb* in standard liquid culture. The dashed line indicates the limit of detection. The frontline anti-TB drug isoniazid was used as a control. (**B**) Impact of Bio-AMS on growth of *Mtb* cultured on different carbon sources. Relative growth was calculated by dividing OD_580_ (optical density at 580 nm) of the culture treated with Bio-AMS by the OD_580_ of the culture in the absence of Bio-AMS. (**C**) Impact of Bio-AMS on intracellular *Mtb* in mouse macrophages. Mouse bone marrow–derived macrophages were infected with *Mtb* in vitro, and the culture was treated with Bio-AMS or dimethyl sulfoxide (DMSO) vehicle 24 hours after infection. Data are representative of two independent experiments each with three replicates and are means ± SEM (**P < 0.01, Student’s *t* test).

We first determined whether inhibition of BPL was sufficient to kill *Mtb*. Isoniazid, a first-line drug that inhibits the synthesis of mycolic acids (*20*), was used as a control. In biotin-free medium, the BPL inhibitor Bio-AMS was bactericidal at a concentration of 5 µM, which is about fivefold the minimum inhibitory concentration (MIC) of Bio- AMS, and killed *Mtb* with kinetics similar to that of isoniazid (Fig. 1A). After 10 days of drug exposure, *Mtb* mutants resistant to isoniazid appeared, whereas Bio-AMS continued to reduce *Mtb* colony-forming units (CFU) until viability of the culture was below the limit of detection of 25 CFU/ml (Fig. 1A).

The anti-mycobacterial activity of several small molecules depends strictly on the primary carbon source used to cultivate *Mtb* (*21*). However, changes in the primary carbon source had little impact on the MIC of Bio-AMS (Fig. 1B) or its minimal bactericidal concentration (fig. S1A). Addition of biotin to the growth medium increased the MIC of Bio-AMS by only fourfold (fig. S1B). However, when we analyzed Bio-AMS with *Mtb* that had ceased to replicate because of starvation in phosphate-buffered saline (PBS), we observed no reduction in *Mtb* viability (fig. S1C). In *Mtb*-infected mouse macrophages in vitro, Bio-AMS inhibited growth of *Mtb* in a concentration-dependent manner (Fig. 1C), and a tetrazolium reduction assay did not detect toxicity for mouse macrophages (fig. S1D). Bio-AMS also showed no signs of mitochondrial toxicity (fig. S2).

### Emergence of *Mtb* resistance to chemical BPL inactivation

**Fig. 2:**
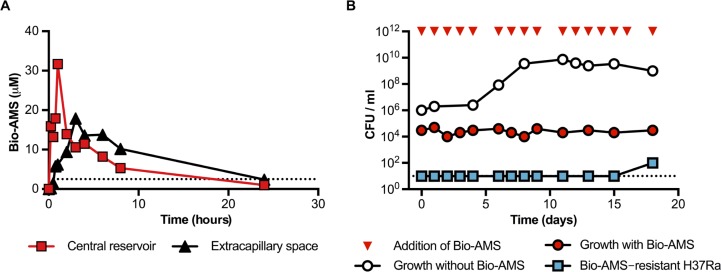
**Emergence of Bio-AMS–resistant *Mtb* strains.** (**A**) Frequency of the emergence of spontaneous resistance in *Mtb* cultured on standard solid medium in the presence of Bio-AMS at concentrations of 10×, 25×, and 50× the MIC. Isoniazid was used as a control. (**B**) Activity of Bio-AMS against *Mtb* strain H37Rv transformed with a multicopy plasmid expressing the *Mtb* protein Rv3406. The *Mtb* H37Rv strain contained either the vector control (pTE-mcs) or the Rv3406 expression plasmid pGMEHPtb38- rv3406. One spontaneously resistant isolate (Bio-AMS-5R1) was included as a control. (**C**) Saturation curve used to determine the kinetic parameters for Bio-AMS oxidation by the *Mtb* protein Rv3406. Left: Data of initial velocity (*v*0) versus concentration of Bio-AMS were fitted by nonlinear regression to the Michaelis-Menten equation. Right: Time course in milliabsorbance units (mAU) showing Rv3406-catalyzed formation of UV active metabolite 3 at 254 nm, as monitored by high-performance liquid chromatography.

*Mtb* mutants that were resistant to Bio-AMS were isolated with a frequency of ~1 in 10^7^ CFU when Bio-AMS was present at a concentration of 10× MIC (Fig. 2A). This frequency decreased to less than 1 in 10^8^ at 25× MIC, and we did not isolate any drug-resistant *Mtb* from 10^8^ CFU at 50× MIC of Bio-AMS. For isoniazid, we observed a resistance frequency of about 1 in 2.5 × 10^6^ CFU, which did not vary much with drug concentration. The Bio-AMS–resistant *Mtb* clones expressed wild-type amounts of BPL (fig. S3A) and did not carry mutations in *rbirA*, the gene encoding BPL. Whole-genome sequencing revealed that all resistant *Mtb* isolates contained mutations in *rv3405c*, most of which were predicted to inactivate *rv3405c*; three strains harbored mutations only in *rv3405c* (table S1). Rv3405c is a transcriptional repressor that controls *rv3406* in the *Mycobacterium bovis* Bacille Calmette-Guerin (BCG) strain (*22*). When we compared mRNAs from Bio-AMS–resistant *Mtb* isolates with those from wildtype *Mtb*, we detected a marked change in *rv3406* (table S2), which resulted in overexpression of Rv3406 protein (fig. S3B). *Mtb* carrying an Rv3406 overexpression plasmid (pGMEH-Ptb38-rv3406) was 64-fold more resistant to Bio-AMS than was wild-type *Mtb* (Fig. 2B). We concluded that overexpression of Rv3406 was sufficient to induce resistance to Bio-AMS in *Mtb*.

Rv3406 is a dioxygenase that oxidizes 2-ethylhexyl sulfate (2-EHS; fig. S3C), and its activity requires nonheme iron (II) and α-ketoglutarate (*23*). To test whether Rv3406 could also oxidize Bio-AMS, we incubated Bio-AMS with recombinant Rv3406 and α-ketoglutarate. This resulted in the time-dependent formation of product that absorbed ultraviolet (UV) light with a λ_max_ of 254 nm and a molecular mass of 265 Da (Fig. 2C), both consistent with formation of adenosine 5′-aldehyde 3 (fig. S3D). This compound likely arises through oxidation of Bio-AMS at C-5′ of the nucleoside and spontaneous disproportionation of the resultant intermediate hemiaminal 2 into aldehyde 3 and *N*-(biotinoyl) sulfamide 4 (fig. S3D). Rv3406 thus oxidized Bio-AMS in the same manner as it oxidizes an alkyl sulfate. This mechanism for enzymatic reactions of alkyl sulfates catalyzed by α-ketoglutarate–dependent dioxygenases is well established (*23*), and the identity of metabolite 4 was confirmed with an authentic standard (fig. S3E). Steady-state kinetic analysis revealed that Bio-AMS is a poor substrate of Rv3406 and has a 48-fold higher *K*_M_ value and nearly 1300-fold lower *k*_cat_ value than 2-EHS (table S3). Nevertheless, degradation of Bio-AMS was dependent on the enzymatic activity of Rv3406 because it only occurred in the presence of α-ketoglutarate (Fig. 2C). Furthermore, we found that the amount of Bio-AMS in *Mtb* was inversely correlated with expression of Rv3406 (fig. S4A). Consistent with the in vitro data, we also measured higher amounts of *N*-(biotinoyl)sulfamide 4 when Rv3406 expression was increased (fig. S4B). Collectively, these results demonstrated that the most frequent mechanism of spontaneous resistance to chemical inactivation of BPL in *Mtb* was enzymatic cleavage of Bio-AMS.

### BPL as a target to control *Mtb* infection in mice

In mice, Bio-AMS was rapidly eliminated, in part due to hydrolysis at the acyl-sulfamide linkage (figs. S5 and S6 and table S4). In addition, the dose of Bio-AMS required to achieve concentrations above the MIC for a substantial fraction of the dosing interval was not tolerated by mice (table S5). We therefore used a hollow fiber system to evaluate the impact of fluctuating Bio-AMS concentrations on growth of *Mtb* together with a genetic approach to determine the consequences of inactivating *Mtb* BPL during infection of mice.

In the hollow fiber culture system, medium is pumped from a central reservoir through a cylindrical bioreactor filled with tubular, semipermeable membrane fibers. By manipulating the flow rate through the system, one can increase or decrease the drug concentration to which the bacteria are exposed, creating defined, dynamic PK drug profiles (*24*). The fiber pore size (20 kDa) ensures that the bacteria introduced into the hollow fiber cartridge are retained in the extracapillary space. A log-phase culture of the *Mtb* strain H37Ra was introduced into the cartridge, allowed to grow in 7H9 medium for 2 days, and then challenged with the BPL inhibitor Bio-AMS. The *C*_max_ was chosen to maintain concentrations above MIC90 for most of the dosing interval. The clearance flow rate was set to achieve a half-life for Bio-AMS of between 9 and 10 hours, which is a typical half-life of several antibiotics in clinical use (*25*, *26*). The PK profiles of Bio-AMS were measured in both the central reservoir and the extracapillary space. PK profiles taken on days 0 and 14 showed *C*_max_ values of _~_32 µM in the central reservoir and ~17 µM in the extracapillary space (Fig. 3A). Viability of *Mtb* H37Ra was monitored by CFU enumeration, which showed that Bio-AMS prevented growth in the hollow fiber system (Fig. 3B). The culture remained free of *Mtb* H37Ra mutant resistant strains after exposure to Bio-AMS for 15 days, but mutant resistant strains did emerge at around day 18 (Fig. 3B). The appearance of Bio-AMS–resistant *Mtb* H37Ra strains suggested that the PK profile analyzed here might have led to a higher frequency of resistance than observed in selection on agar plates.

**Fig. 4:**
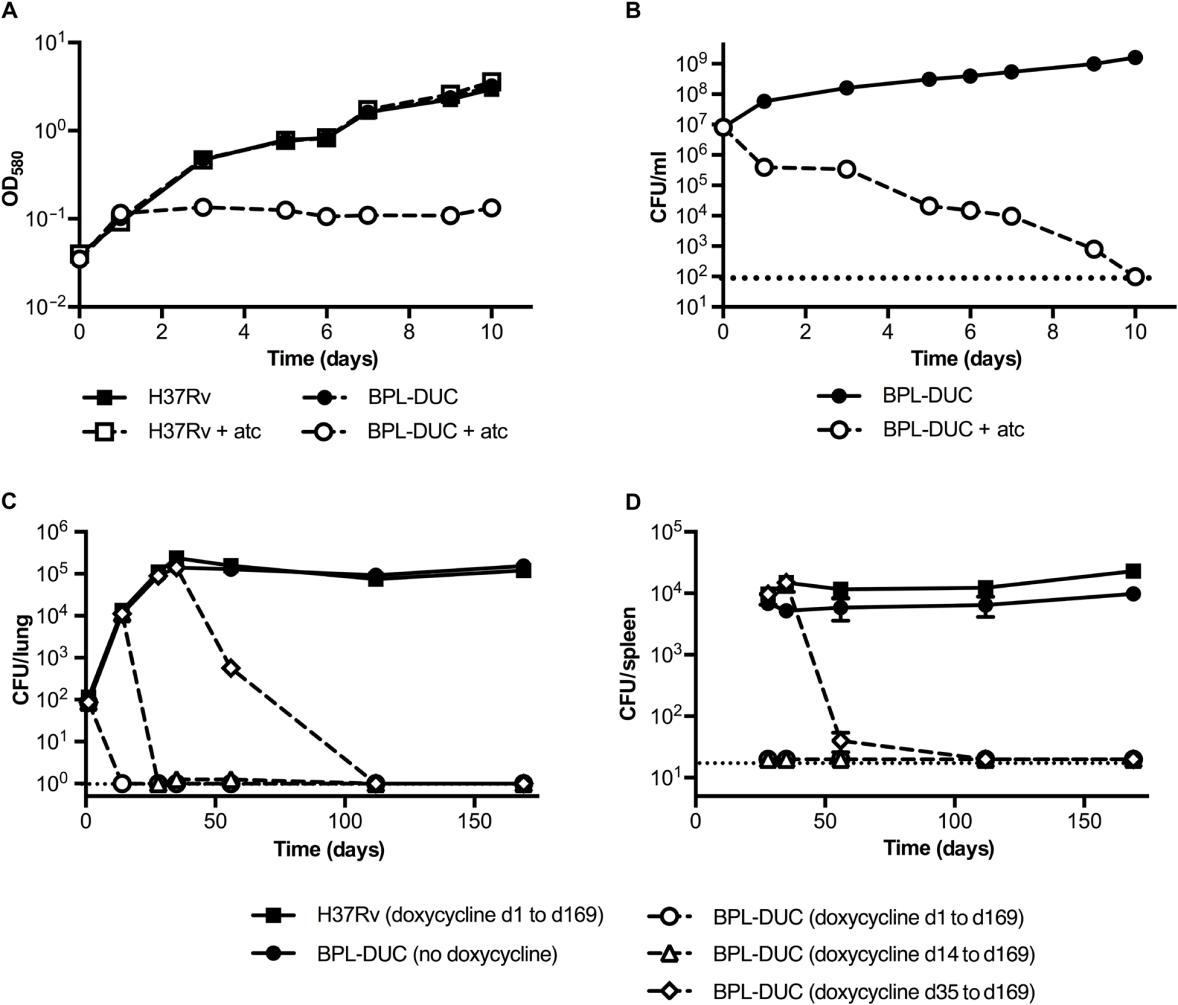
**Effect of depleting BPL on *Mtb* in vitro and in vivo in mice.** (**A** and **B**) Impact of BPL depletion on growth (A) and survival (B) of the *Mtb* BPL-DUC strain in standard liquid culture medium. Growth and survival were monitored using measurements of OD and counting of CFU, respectively. (**C** and **D**) CFU isolated from mouse lungs (C) and mouse spleens (D) after infection of mice with the *Mtb* H37Rv strain or the *Mtb* mutant BPL-DUC strain. Mice infected with the *Mtb* H37Rv strain received doxycycline. Data are representative of three (A and B) or two (C and D) independent experiments each with three (A and B) or four (C and D) samples per time point. Data are means ± SEM.

Next, we used a previously described dual control switch (*27*, *28*) to construct an *Mtb* mutant strain, BPL-DUC, in which anhydrotetracycline (atc) induces both transcriptional silencing of *birA* and proteolytic inactivation of BPL (fig. S7). Exposure of the BPL-DUC strain to atc (i) depleted BPL below the limit of detection of immunoblots within 24 hours (fig. S8A), (ii) caused a decrease in biotinylated proteins (fig. S8B), (iii) prevented growth of BPL-DUC *Mtb* (Fig. 4A), and (iv) decreased viability of BPL-DUC *Mtb* (Fig. 4B). C57BL/6 mice were infected with BPL-DUC *Mtb*, and the mice were fed chow containing doxycycline from the beginning of the infection, 14 days after initial infection, 35 days after initial infection, or not at all (Fig. 4, C and D). Mice that were infected with *Mtb* H37Rv and received doxycycline-supplemented food served as a control. As early as 14 days after infection, we failed to recover CFU for BPL-DUC *Mtb* from the lungs of mice that received doxycycline throughout the infection (Fig. 4C). Similarly, when doxycycline-supplemented food was given on day 14 after initial infection, we did not recover any CFU from the lungs 2 weeks later. When BPL was inactivated during the chronic phase of infection by switching to doxycycline-supplemented food on day 35 after infection, we observed a decrease in CFU of more than two logs by day 56, and lungs were free of CFU by the next time point (day 112 after infection) (Fig. 4C). On day 169 after infection, we did not recover CFU from the lungs of any of the groups that received doxycycline-supplemented food (Fig. 4C). Decreases in CFU that occurred after doxycycline treatment were accompanied by reduced lung tissue pathology (fig. S9). Depleting BPL had a similar impact on CFU in the spleen as it had in the lung (Fig. 4D).

### Importance of *Mtb* protein biotinylation for the activity of rifampicin, isoniazid, and ethambutol

**Fig. 5:**
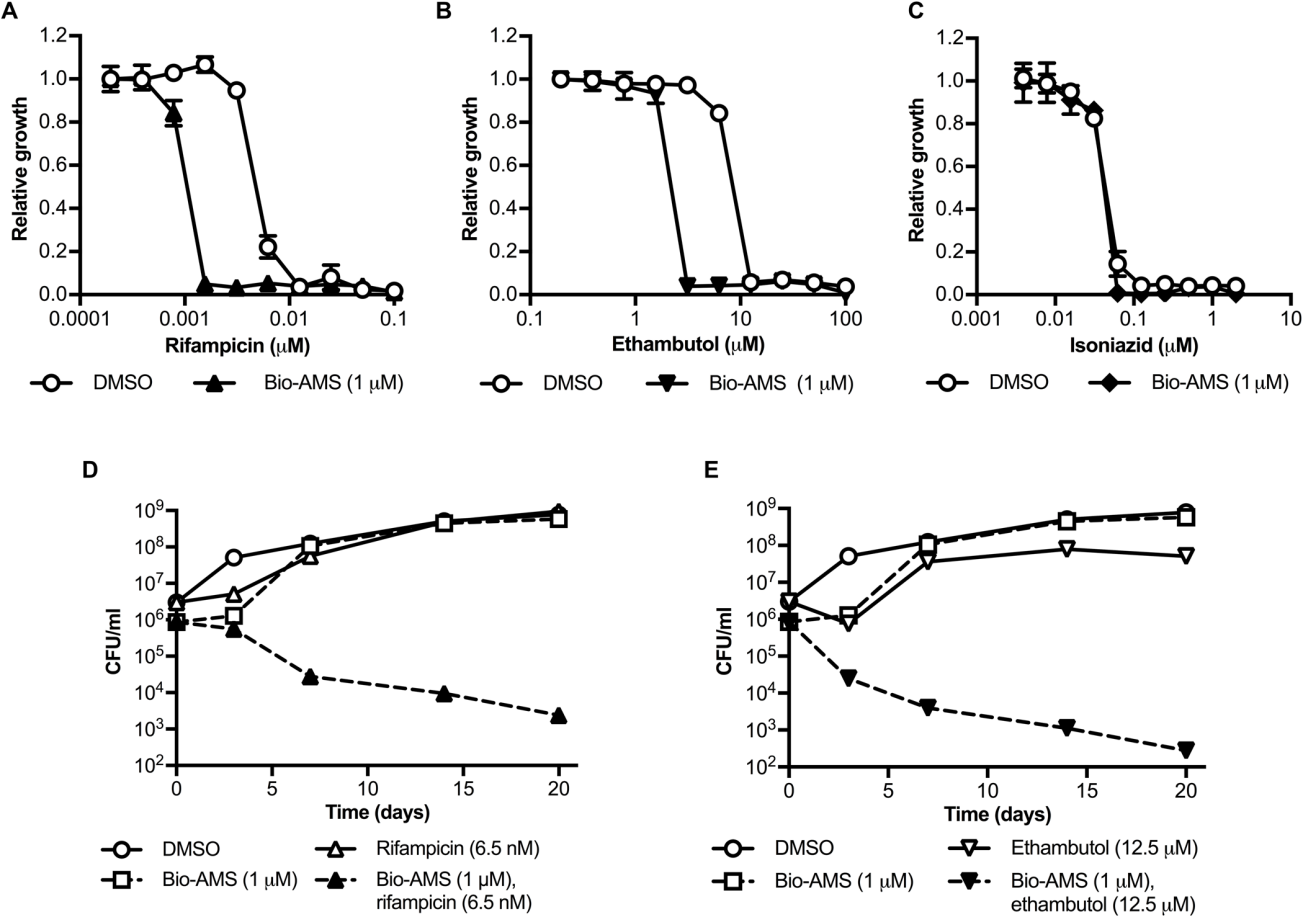
**Bio-AMS enhances the activity of rifampicin and ethambutol in vitro.** (**A** to **E**) Impact of Bio-AMS on the activities of the TB drugs rifampicin (A and D), ethambutol (B and E), and isoniazid (C). The *Mtb* H37Rv strain was grown in standard liquid culture with Bio-AMS (1 µM in DMSO) or DMSO alone for 3 days, after which the bacteria were exposed to the other anti-TB drugs. Data are representative of two independent experiments with three samples per time point. Data are means ± SEM.

The cell envelope of mycobacteria is a highly selective barrier that contributes greatly to the intrinsic drug resistance of *Mtb* (*3*, *4*). The integrity of this envelope requires fatty acids and lipids, which depend on biotinylated ACC enzymes for synthesis. We therefore analyzed how inhibition of protein biotinylation affected *Mtb*’s acid-fastness (which we used as an indicator of *Mtb*’s cell envelope composition) (*29*) and its susceptibility to the anti-TB antibiotics rifampicin, isoniazid, and ethambutol. First, we treated *Mtb* with a lethal dose of Bio- AMS and analyzed the cell envelope using acid-fast staining before and after the amounts of biotinylated proteins were reduced (fig. S10). We detected an increase in acid-fast–negative bacteria on day 5 after exposure (when biotinylated proteins just began to decrease); after 10 days of exposure to Bio-AMS, most bacteria no longer stained as acid-fast (fig. S10). Next, we measured whether a sublethal dose of Bio-AMS changed the susceptibility of *Mtb* to rifampicin, isoniazid, and ethambutol. Because it took several days before exposure to Bio- AMS affected *Mtb*’s pattern of protein biotinylation, we first grew *Mtb* with and without Bio-AMS for 3 days and then exposed these cultures individually to rifampicin, isoniazid, and ethambutol—in each case, with or without Bio-AMS. Sublethal doses of Bio-AMS reduced the MIC of rifampicin and ethambutol but left the MIC of isoniazid unchanged (Fig. 5, A to C). We then determined whether Bio-AMS could also enhance the bactericidal activity of rifampicin and ethambutol. For these experiments, we used Bio-AMS at 1 µM, rifampicin at 6.5 nM, and ethambutol at 12.5 µM. As expected, none of the compounds were bactericidal at these concentrations when used individually (Fig. 5, D and E). However, the combined use of either rifampicin or ethambutol with Bio-AMS killed more than 99.7 and 99.9% of the *Mtb* inoculum, respectively, after 20 days of treatment.

We next asked whether the increased potency of rifampicin and ethambutol caused by Bio-AMS could be replicated by limiting access to biotin. We grew the biotin auxotroph *Mtb* ∆*bioA* in medium with defined biotin concentrations and measured the MIC of rifampicin and ethambutol. We found that decreasing concentrations of biotin decreased the MIC of rifampicin but left the MIC of ethambutol unchanged (fig. S11, A and B). When we compared the amount of rifampicin that accumulated in wild-type *Mtb* and *Mtb* ∆*bioA*, we found increased amounts of rifampicin in *Mtb* ∆*bioA* when the mutant strain was grown in medium with low concentrations of biotin (fig. S11C). Thus, Bio-AMS improved the potency of rifampicin by interfering with synthesis of a normal cell envelope, as indicated by the reduction in acid-fastness, which, in turn, facilitated rifampicin uptake. In addition, Bio-AMS decreased the MIC of ethambutol by an as yet to be determined mechanism.

### Impact of interfering with protein biotinylation on the activity of rifampicin during *Mtb* infection

**Fig. 6:**
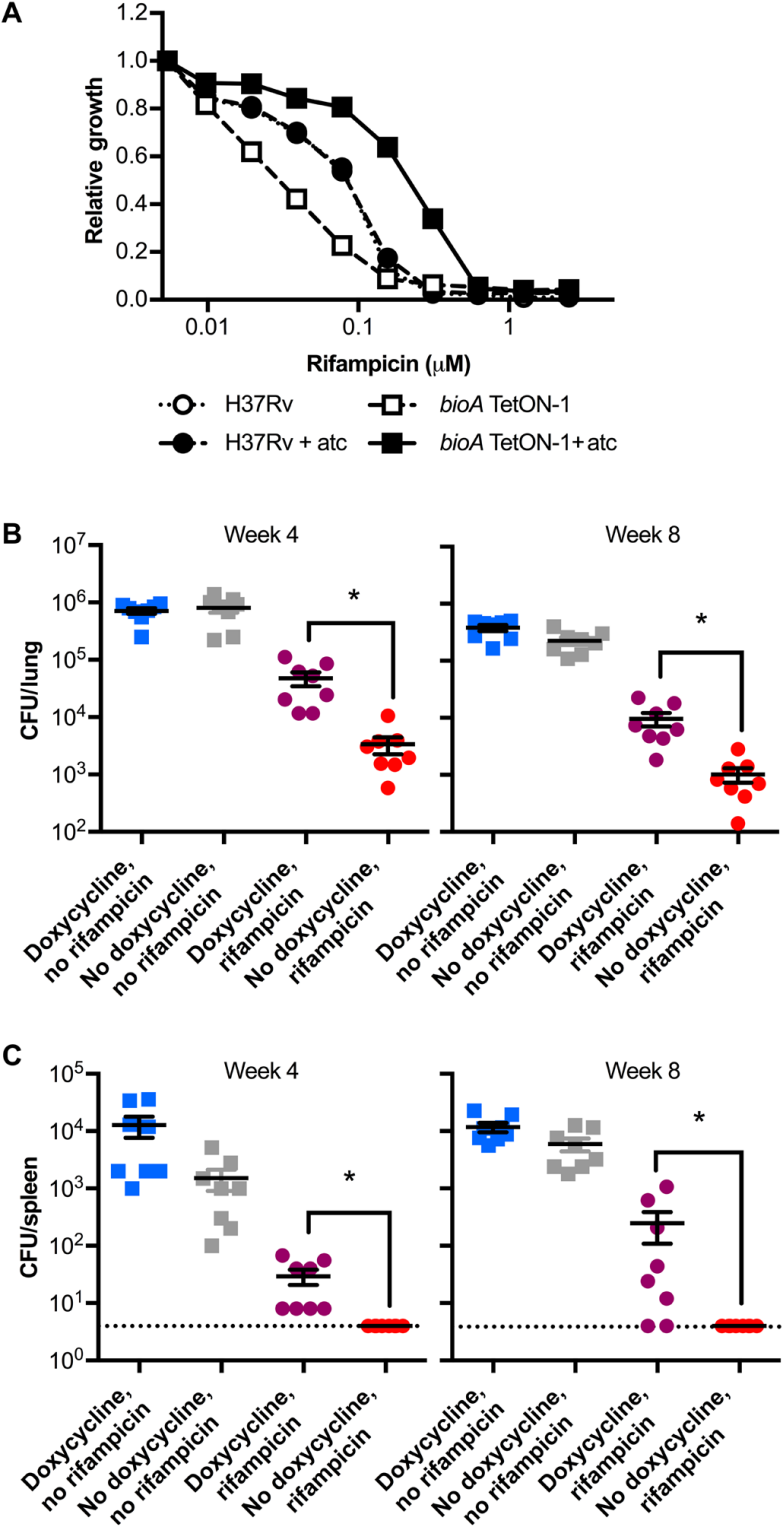
**Partial inhibition of biotin synthesis increases susceptibility of *Mtb* to rifampicin in mice.** (**A**) Susceptibilities of the *Mtb* H37Rv strain and the *Mtb* mutant *bioA* TetON-1 strain to rifampicin in biotin-free medium with and without atc. (**B**) Number of CFU isolated from the lungs of mice infected with the *bioA* TetON-1 *Mtb* strain. Mice were fed either doxycycline-containing (blue and purple) or doxycycline-free (gray and red) chow throughout the experiment. Rifampicin was administered 3 weeks after initial infection to one group of mice that received doxycycline (purple) and one group that had not received doxycycline (red). All samples were collected at 4 or 8 weeks after treatment with rifampicin was initiated. (**C**) Number of CFU isolated from the spleens of mice described in (B). Data in (B) and (C) are for *n* = 8 mice for each time point. Data are means ± SEM (*P < 0.05, Mann-Whitney test).

Together with isoniazid, rifampicin forms one of the cornerstones for treatment of drug-sensitive TB. We therefore sought to determine whether sublethal interference with *Mtb* protein biotinylation could also improve the potency of rifampicin in mice during infection with *Mtb*. It would have been very difficult to answer this question using BPL-DUC *Mtb* because this mutant strain is cleared too rapidly from mice that received doxycycline. In previous work, however, we had constructed a panel of *bioA*-TetON *Mtb* mutants, in which transfer from medium containing atc or doxycycline to atc/doxycycline-free medium silenced expression of BioA to different degrees (*30*). One of these mutants, *bioA* TetON-1, expresses ~1000% BioA with atc and _~_5% BioA without atc compared to wild-type *Mtb* but grows almost as well as wild-type *Mtb* in biotin-free medium (*30*). We therefore measured rifampicin sensitivity of the *bioA* TetON-1 mutant and found the mutant to be more sensitive than wild-type *Mtb* to rifampicin without atc and more resistant than wild-type *Mtb* with atc (Fig. 6A).

Next, we infected C57BL/6 mice with *bioA* TetON-1 in aerosolized form and divided the mice into two groups, one of which received doxycycline-supplemented chow and the other was fed doxycycline-free chow. Twenty-one days after infection, rifampicin was administered by oral gavage 5 days a week at a dose of 10 mg/kg for either 4 or 8 weeks. At the end of rifampicin treatment, the mice were kept without rifampicin for 3 days, and then CFU were determined in lungs and spleens. In agreement with our previous experiments (*30*), there was no difference in bacterial CFU between the two groups of mice on doxycycline-supplemented or regular chow in the absence of rifampicin, suggesting that this mutant strain could sustain infection in mice even after expression of BioA was reduced (Fig. 6, B and C). Both treatment groups showed progressive reduction of CFU after rifampicin treatment compared to the untreated control groups at 4 and 8 weeks after treament with rifampicin was initiated. However, fewer CFU (*P* < 0.05) were obtained from the lungs of infected mice when BioA expression was reduced because of the lack of doxycycline compared to when BioA was expressed constitutively (Fig. 6B). Similar results were obtained for CFU counts in the spleens of these mice (Fig. 6C). Without doxycycline, treatment with rifampicin alone was sufficient to kill *Mtb bioA* TetON-1 in mouse lungs by more than 200-fold within 4 weeks. This efficacy is similar to that achieved by combining rifampicin with isoniazid (*31*, *32*).

To test whether doxycycline could have indirectly changed exposure to rifampicin, we performed a drug-drug interaction study. Rifampicin was administered with and without doxycycline to uninfected mice, and then we measured both doxycycline and rifampicin plasma concentrations after a single rifampicin dose and after seven daily doses, that is, at steady state when the overall intake of the drug was in dynamic equilibrium with its elimination. Seven daily doses of rifampicin caused a small but statistically significant decrease (_~_30%; *P* < 0.05) in doxycycline exposure over the 8-hour period during which plasma concentrations were measured. However, rifampicin exposure was not affected by co-administration of doxycycline (table S6). In addition, drug distribution studies with doxycycline administered in the diet to *Mtb*-infected rabbits showed that doxycycline accumulated in lung lesions relative to plasma, with lesion/plasma ratios ranging from about 4 to 6 (table S7). Accumulation of doxycycline in tissues was independent of the doxycycline concentration in the diet [200 or 400 parts per million (ppm)]. Thus, doxycycline concentrations in *Mtb* lung lesions in infected rabbits were at least as high as those measured in plasma (table S7).

## DISCUSSION

TB remains a major killer in the developing world. Continued improvement in TB chemotherapy, including the development of new drugs, will be required to eradicate this infectious disease. New TB drugs should be ideally active against both drug-sensitive and drug-resistant *Mtb* and should shorten the treatment time period. *Mtb* BPL has been shown to be susceptible to chemical inhibition by Bio-AMS, which can prevent growth of both drug-sensitive and drug-resistant *Mtb* in vitro (*17*). This finding suggested that *Mtb* BPL may be a potentially attractive target for TB drug development, but its importance for bacterial viability under different physiological conditions and during *Mtb* infection of animal models remained to be determined. It was also unclear whether inhibition of BPL affected the potency of current first-line drugs and how it might affect the length of treatment. By answering these questions, we sought to further evaluate the potential of Bio-AMS as a lead compound and BPL as a viable target for TB drug development.

Here, we report that chemical inhibition of BPL was bactericidal for *Mtb* during growth on different carbon sources (Fig. 1, A and B, and fig. S1A), prevented growth in mouse macrophages (Fig. 1C), and resulted in a low number of drug-resistant mutant *Mtb* strains (Fig. 2A). Inhibition of BPL also prevented growth of *Mtb* in a hollow fiber culture system that simulated fluctuating Bio-AMS concentrations typically observed in in vivo systems (Fig. 3) and showed synergy with other TB drugs (Fig. 5, A and B). To determine the consequences of BPL inhibition during *Mtb* infection of mice, we constructed an *Mtb* BPL-DUC strain (fig. S7) that enabled rapid depletion of BPL (fig. S8A). Characterization of this mutant confirmed our conclusions from chemical BPL inactivation experiments and revealed that depletion of BPL was sufficient to rapidly kill *Mtb* during acute and chronic infection in mice (Fig. 4, C and D, and fig. S9). Depletion of BPL eradicated *Mtb* not only during acute infections but also when it was initiated on day 35 after infection when the bacteria had already established a chronic infection. This is in contrast to both the lack of Bio-AMS activity against nonreplicating *Mtb* in vitro and the potency of isoniazid, which is higher if administered during the acute phase (for example, beginning on day 3 after infection) rather than during the chronic phase (for example, beginning on day 28 after infection) (*33*). The fact that BPL inactivation efficiently killed *Mtb* during the chronic phase of infection in mice is consistent with data demonstrating that *Mtb* is still replicating during this stage of the infection, although the CFU count remains stable (*34*). Moreover, it suggests that BPL might also be required for ACC enzymes to synthesize the building blocks for cell wall remodeling that seems to occur during chronic infection (*35*). Together, these experiments demonstrate that interfering with *Mtb*’s ability to biotinylate proteins increases sensitivity to rifampicin, and suggest that Bio-AMS or other inhibitors targeting biotin metabolism may be excellent drug candidates.

One limitation of our work characterizing *Mtb* BPL is that it has not yet resulted in a BPL inhibitor with sufficient bioavailability. Notably, susceptibility to cleavage at the biotin-adenosine linker both contributes to the limited bioavailability of Bio-AMS and is the primary mechanism of spontaneous resistance of *Mtb* to this compound. Ongoing experiments that aim to improve the bioavailability of Bio- AMS therefore should focus on modifications that enhance the stability of the acyl-sulfamide linker region of the molecule connecting the biotin and nucleoside moieties or on analogs where the acyl-sulfamide is replaced with an equivalent functional group. Using this approach, it may be possible to not only improve bioavailability but also to further decrease the frequency of emergence of drug-resistant *Mtb* mutants by overcoming Rv3406-mediated destruction of Bio-AMS. The enzymes that cleave Bio-AMS in mice also remain unknown; however, we were able to identify the alkyl sulfatase Rv3406 as the enzyme responsible for Bio-AMS degradation in *Mtb*. Neres *et al*. (*36*) have shown that an unrelated class of 2-carboxyquinoxalines that target DprE1 are also inactivated by Rv3406 but via an alternative mechanism whereby the compounds mimic the substrate α-ketoglutarate. This suggests that the Rv3406 enzyme of *Mtb* may have a more general role in xenobiotic metabolism.

It also remains to be determined how fluctuating Bio-AMS concentrations influence the frequency of emergence of Bio-AMS–resistant *Mtb* mutants. It will be important to define how PK parameters influence the frequency and type of mutations leading to Bio-AMS resistance in *Mtb*. Nevertheless, when using Bio-AMS at a peak concentration and half-life similar to those of other clinically used cell wall–targeting antibiotics, the simulations in a hollow fiber culture system revealed that incomplete chemical inhibition of BPL achieved static growth inhibition of *Mtb* across the dosing interval. Finally, as has been discussed in detail elsewhere (*37*), the mouse model of TB has its shortcomings. This includes the fact that the pathology resulting from *Mtb* infection in mice is not fully representative of the pathology observed in humans suffering from active TB and that mice do not develop human-like latent TB infection (*37*). How inactivation of BPL affects growth and survival of *Mtb* in the spectrum of granulomas observed in humans thus remains to be determined.

Successful therapy of TB depends on drug combinations, and new drugs should ideally synergize with existing frontline drugs. Partial inhibition of BPL using sublethal concentrations of Bio-AMS increased the potency of rifampicin and ethambutol, revealing an attractive feature of BPL as a target for TB drug development (Fig. 5). Limiting *Mtb*’s access to biotin also allowed us to determine how reducing *Mtb* protein biotinylation affected the potency of rifampicin during *Mtb* infection of mice. This finding showed that partial inhibition of *Mtb* protein biotinylation, which by itself did not reduce growth of *Mtb* in mouse lungs, accelerated killing of *Mtb* by rifampicin in infected mice (Fig. 6, B and C). Notably, the potency we observed for treatment of an *Mtb* mutant with impaired protein biotinylation by rifampicin alone was similar to that reported for treatment of wild-type *Mtb* using a combination of rifampicin and isoniazid (*31*, *32*).

Rifampicin and isoniazid are two of the most important drugs for treatment of TB. Isoniazid has the most potent bactericidal activity during the early treatment phase, and rifampicin is most effective in preventing relapse (*38*–*40*). The importance of the isoniazid and rifampicin combination is evidenced by the fact that TB caused by *Mtb* that is resistant to isoniazid and rifampicin is classified as MDRTB, irrespective of resistance to other drugs. Inactivation of BPL resembles inhibition of InhA, the target of isoniazid, in that blocking both of these *Mtb* enzymes interferes with cell envelope biosynthesis and kills growing *Mtb* rapidly both in vitro and during in vivo infection. Potentially, drugs targeting *Mtb* BPL might be as effective as isoniazid for treating TB and could help to further shorten TB chemotherapy by improving the potency of rifampicin.

## MATERIALS AND METHODS

### Study design

The overall objective of this study was first to provide a more thorough evaluation of the previously described BPL inhibitor Bio-AMS and then to further validate *Mtb* BPL as a target for TB drug development. Bio-AMS was characterized with respect to its activity under a variety of *Mtb* growth conditions, its PK properties, its ability to induce emergence of drug-resistant mutants, and its interactions with existing TB drugs. Genetic approaches were applied to evaluate BPL as a target for TB drug development by determining the consequences of depleting BPL in vitro and during infection of mice. Animals were randomly allocated into groups and were identifiable with respect to their treatment during the experiments. All studies were carried out in accordance with the *Guide for the Care and Use of Laboratory Animals* of the National Institutes of Health, with approval from the Institutional Animal Care and Use Committee (IACUC) of the New Jersey Medical School, Rutgers University, Newark, or the IACUC of Weill Cornell Medical College. Animals were maintained under pathogen-free conditions and fed water and chow ad libitum; all efforts were made to minimize suffering or discomfort. All experiments with *Mtb* were carried out in a biosafety level 3 facility and approved by the relevant institutional biosafety committees.

### Materials and reagents

Middlebrook 7H9 medium, 7H10 medium, and Middlebrook OADC (oleic albumin dextrose catalase) growth supplement were from Difco. Hygromycin (Thermo Fisher Scientific), kanamycin (Sigma-Aldrich), and zeocin (Invitrogen) were used at a concentration of 50, 25, and 25 μg/ml, respectively. Anti-BPL antiserum was generated using purified BPL protein by Covance and used at a dilution of 1:2500. Anti-rv3406 antiserum was a gift from L. Mendonça-Lima and used at a dilution of 1:1000. Strains are listed in table S8.

### Antibacterial activity measurements

MIC measurements were performed as described (*18*). CFUs were used as a readout to assess bactericidal activities in liquid culture and during infections of bone marrow–derived macrophages. Carbon sources were used at a concentration of 0.1%. PBS starvation was used as a model for nonreplicating bacteria, as described previously (*28*).

### *Mtb* resistance to Bio-AMS

About 10^8^ bacteria were cultured on 7H10 agar plates containing drug at a concentration of 10×, 25×, or 50× the MIC. Frequency of resistance was calculated as the number of CFU per 10^8^ bacteria plated. mRNA analyses of Bio-AMS–resistant strains were performed as described in our previous work (*41*).

### Mouse infection with *Mtb* BPL-DUC and *bioA* TetON-1 mutant strains

Four- to 6-week-old female C57BL/6 mice were infected with ~100 CFU of BPL-DUC strain and divided into various groups. The control group was maintained on a regular diet, whereas test groups received doxycycline rodent chow (2000 ppm; Research Diets) when indicated. At each time point, four mice per group were sacrificed, the lung and spleen were homogenized in PBS, and dilutions were cultured on antibiotic-free agar plates. C57BL/6 mice were infected with *bioA* TetON-1 as for BPL-DUC. The control group received doxycycline throughout the experiment; the test group received the regular chow. Rifampicin was administered at a dosage of 10 mg/kg by oral gavage 5 days a week. Eight mice from each group were sacrificed 4 and 8 weeks after rifampicin treatment, and organs were processed as described above.

### PK profiling

Groups of four mice received Bio-AMS formulated in 0.9% saline according to the following dosing scheme: 5 mg/kg via the intravenous route, 25 mg/kg via the intraperitoneal route, and 25 mg/kg via the oral route. Blood samples were collected in heparinized tubes predose and 5 min, 15 min, 30 min, 1 hour, 1.5 hours, 3 hours, 5 hours, and 8 hours post-dose after intravenous and intraperitoneal injections, and pre-dose and 5 min, 30 min, 1 hour, 3 hours, 5 hours, and 8 hours after oral gavage. Blood samples were centrifuged to recover plasma and quantify Bio-AMS and its major metabolites by highpressure liquid chromatography coupled to tandem mass spectrometry, as described in the Supplementary Materials.

### Tolerability

Groups of three mice received Bio-AMS (50, 100, 250, or 500 mg/kg) via intraperitoneal injection at 4 ml/kg. They were observed continuously for the first half-hour after injection and then at 24 hours after injection.

### Biochemical characterization of Rv3406

His-tagged Rv3406 was overexpressed in BL21 (DE3) *E. coli,* purified as described (*23*). Enzymatic activities were established as described in the Supplementary Materials.

### Intrabacterial PKs

A previously described (*28*) experimental setup was used to study accumulation of Bio-AMS and its degradation products inside *Mtb*. Briefly, *Mtb*-laden filters were grown in Middlebrook 7H10 agar plates for 5 days, followed by exposure to Bio-AMS for 18 hours in GAST medium containing 25 µM Bio-AMS or an equivalent amount of DMSO. After 18 hours, the filters were incubated on GAST medium for 24 hours without any antibiotics, and samples were collected and processed as described in the Supplementary Materials.

### Activity of Bio-AMS in a hollow fiber culture system

*Mtb* H37Ra (American Type Culture Collection 25177) was grown in Middlebrook 7H9 medium supplemented with OADC for 4 days at 37˚C. High-flux polysulfone hollow fiber cartridges (C2011, FiberCell Systems Inc.) were equilibrated for 3 days with 7H9 OADC. Initial PK data were obtained for Bio-AMS diluted in 7H9 OADC and infused with a syringe pump into the hollow fiber system. A clearance flow rate that simulated a Bio-AMS half-life of 9 to 10 hours in the extracapillary space was used. To determine the susceptibility of H37Ra to Bio-AMS under defined PK parameters, a new hollow fiber cartridge pre-equilibrated with 7H9 OADC was inoculated with 20 ml of a bacterial suspension of 10^4^ to 10^6^ CFU/ml. The culture was allowed to establish in the cartridge extracapillary space under a constant flow of 7H9 OADC (30 ml/hour) for 2 days before the first Bio-AMS infusion. To achieve a *C*_max_ of at least 9 µM in the extracapillary space of the hollow fiber system, 1.42 mg of Bio-AMS was infused for 60 min at a flow rate of 30 ml/hour. This infusion was repeated on an almost daily basis for 18 days. Bio-AMS concentrations in hollow fiber samples were quantified by mass spectrometry, except that only 1 µl volumes of extracted samples were injected. PK analysis of drug concentrations attained in both the central reservoir and extracapillary space was performed on days 0 and 14.

### Statistical analysis

Averages were used as a measure of central tendency. Data from continuous variables were analyzed using Mann-Whitney and Student’s *t* tests. Differences with *P* ≤ 0.05 were considered statistically significant. All statistical analyses were performed using GraphPad Prism 7.0 (GraphPad Software Inc.).

## SUPPLEMENTARY MATERIALS

www.sciencetranslationalmedicine.org/cgi/content/full/10/438/eaal1803/DC1

Materials and Methods

Fig. S1. Bio-AMS kills *Mtb* in medium with different carbon sources and is not acutely toxic to mouse macrophages.

Fig. S2. Evaluation of mitochondrial toxicity.

Fig. S3. Emergence of *Mtb* mutants resistant to Bio-AMS.

Fig. S4. Quantification of *Mtb*-associated Bio-AMS and biotin sulfonamide.

Fig. S5. PK profiles and metabolism of Bio-AMS in mice.

Fig. S6. Putative Bio-AMS metabolic and degradation pathways.

Fig. S7. Construction of the *Mtb* BPL-DUC strain.

Fig. S8. Impact of atc on BPL expression and protein biotinylation.

Fig. S9. Histopathology of lungs infected with the *Mtb* BPL-DUC strain.

Fig. S10. Bio-AMS treatment inhibits protein biotinylation and results in loss of *Mtb* acid-fastness.

Fig. S11. Growth of *Mtb* Δ*bioA* in low concentrations of biotin increases potency of rifampicin but not ethambutol.

Table S1. Whole-genome sequencing of Bio-AMS–resistant *Mtb* strains.

Table S2. Genes whose transcripts changed more than threefold in three Bio-AMS–resistant strains.

Table S3. Kinetic parameters of *Mtb* Rv3406.

Table S4. PK parameters of Bio-AMS after intravenous, intraperitoneal, and oral administration.

Table S5. Tolerability of Bio-AMS at ascending intraperitoneal doses in CD-1 mice.

Table S6. Concentrations of rifampicin and doxycycline in the plasma of CD-1 mice receiving rifampicin alone or rifampicin with doxycycline in the diet after a single dose (10 mg/kg) of rifampicin and at a steady state.

Table S7. Distribution of doxycycline in *Mtb*-infected rabbit lung lesions relative to plasma after administration of doxycycline in chow for 7 days.

Table S8. Strains and plasmids.

## Supplementary Material

Targeting protein biotinylation enhances tuberculosis chemotherapy
