# Supplementary material for: Targeting protein biotinylation enhances tuberculosis chemotherapy
Source: Sci Transl Med. 2018 Apr 25;10(438):eaal1803. doi: 10.1126/scitranslmed.aal1803 (PMC6151865; doi:10.1126/scitranslmed.aal1803)
Supplement: Targeting protein biotinylation enhances tuberculosis chemotherapy [file STM-10-eaal1803-s1.pdf]

Science Translational Medicine
AAAS

www.sciencetranslationalmedicine.org/cgi/content/full/10/438/eaal1803/DC1

Supplementary Materials for

Targeting protein biotinylation enhances tuberculosis chemotherapy

Divya Tiwari, Sae Woong Park, Maram M. Essawy, Surendra Dawadi, Alan Mason, Madhumitha Nandakumar, Matthew Zimmerman, Marizel Mina, Hsin Pin Ho, Curtis A. Engelhart, Thomas Ioerger, James C. Sacchettini, Kyu Rhee, Sabine Ehrt, Courtney C. Aldrich, Véronique Dartois,* Dirk Schnappinger*

*Corresponding author. Email: dis2003@med.cornell.edu (D.S.); dartoiva@njms.rutgers.edu (V.D.)

Published 25 April 2018, *Sci. Transl. Med.* **10**, eaal1803 (2018)
DOI: 10.1126/scitranslmed.aal1803

This PDF file includes:

Materials and Methods
Fig. S1. Bio-AMS kills *Mtb* in medium with different carbon sources and is not acutely toxic to mouse macrophages.
Fig. S2. Evaluation of mitochondrial toxicity.
Fig. S3. Emergence of *Mtb* mutants resistant to Bio-AMS.
Fig. S4. Quantification of *Mtb*-associated Bio-AMS and biotin sulfonamide.
Fig. S5. PK profiles and metabolism of Bio-AMS in mice.
Fig. S6. Putative Bio-AMS metabolic and degradation pathways.
Fig. S7. Construction of the *Mtb* BPL-DUC strain.
Fig. S8. Impact of atc on BPL expression and protein biotinylation.
Fig. S9. Histopathology of lungs infected with the *Mtb* BPL-DUC strain.
Fig. S10. Bio-AMS treatment inhibits protein biotinylation and results in loss of *Mtb* acid-fastness.
Fig. S11. Growth of *Mtb* Δ*bioA* in low concentrations of biotin increases potency of rifampicin but not ethambutol.
Table S1. Whole-genome sequencing of Bio-AMS–resistant *Mtb* strains.
Table S2. Genes whose transcripts changed more than threefold in three Bio-AMS–resistant strains.
Table S3. Kinetic parameters of *Mtb* Rv3406.
Table S4. PK parameters of Bio-AMS after intravenous, intraperitoneal, and oral administration.
Table S5. Tolerability of Bio-AMS at ascending intraperitoneal doses in CD-1 mice.

Table S6. Concentrations of rifampicin and doxycycline in the plasma of CD-1 mice receiving rifampicin alone or rifampicin with doxycycline in the diet after a single dose (10 mg/kg) of rifampicin and at a steady state.
Table S7. Distribution of doxycycline in *Mtb*-infected rabbit lung lesions relative to plasma after administration of doxycycline in chow for 7 days.
Table S8. Strains and plasmids.

MATERIALS AND METHODS

Rifampicin-doxycycline interaction studies

Four groups of 4 CD-1 female mice 4 to 6 weeks of age, weighing 20-22 g, received rifampicin via oral gavage and/or 2000 ppm. The rifampicin formulation was prepared in 40% sucrose, placed in a water-bath sonicator for 10 min followed by probe sonication for 3 min. A microsampling study design was adopted where serial blood samples (40-50 μL) were collected longitudinally from the tail vein of groups of 4 mice. Group 1 received a single dose of 10 mg/kg rifampicin only; group 2 received seven consecutive days of doxycycline-containing chow with a single dose of 10 mg/kg rifampicin on Day 7; group 3 received seven consecutive daily doses of 10 mg/kg rifampicin and group 4 received seven consecutive days of both doxycycline-containing chow and 10 mg/kg rifampicin. Blood samples were collected on the last day of dosing in heparinized tubes, pre-dose and 5 min, 30 min, 1 h, 3 h, 5 h, and 8 h post-dose. Blood samples were centrifuged to recover plasma and quantify rifampicin and doxycycline by LC/MS-MS.

Quantitation of rifampicin and doxycycline in mouse plasma

Rifampicin and doxycycline standards were obtained from Tokyo Research Chemical and Research Products International, respectively. Analytes of interest were extracted by diluting 20 μL of mouse plasma with 20 μL of acetonitrile:water (1:1), 5 μL of 75 mg/mL ascorbic acid, and 180 μL of methanol:acetonitrile (1:1) containing 20 ng/mL of rifampicin-d8 internal standard (IS) for rifampicin and 10 ng/mL Verapamil IS for doxycycline. Rifampicin-d8 and verapamil were obtained from Toronto Research Chemicals, Inc and Sigma-Aldrich respectively. The mixture was vortexed and centrifuged, and 200 μL of the supernatant was recovered for analysis. LC/MS-MS analysis was performed with an Agilent 1260 system coupled to an AB Sciex 4000 Q-trap Mass Spectrometer (positive mode electrospray ionization), and an Agilent column SB-

C8, 2.1 × 30 mm, 3.5 µm, with the column temperature fixed at 24 °C. Mobile phase A was 0.1% formic acid in 100% H_2O and mobile phase B was 0.1% formic acid in 100% acetonitrile. Injection volumes were routinely 2 µL. The Mass Selective Detector was set to MRM (multiple reaction monitoring) mode using positive polarity ionization, monitoring for the ions of interest (*m/z* 823.50/791.60 for rifampicin; *m/z* 445.20/410.10 for doxycycline) and the internal standards (*m/z* 831.50/799.60 for rifampicin-d8 and 455.40/165.20 for verapamil). The lower limit of quantification was 5 ng/mL for rifampicin and 50 ng/mL for doxycycline.

Pharmacokinetic parameters (areas under the curve ($AUC_{[0-t]}$, C_{max}) were calculated from mean concentrations using Microsoft Excel (Office 2010, Microsoft Corp, Redmond, WA). AUCs were calculated using the linear trapezoidal rule. Concentration values below the lower limit of quantification were excluded from the pharmacokinetic evaluation. An unpaired parametric t test was used to compare AUC values, assuming unequal standard deviations.

Penetration of doxycycline from plasma to lung lesions in rabbits

Specific pathogen-free, individually housed female NZW rabbits, weighing 2.2 to 2.6 kg, were used for aerosol infection by *Mtb* strain HN878, as previously described (S. Subbian, L. Tsenova, G. Yang, P. O'Brien, S. Parsons, B. Peixoto, L. Taylor, D. Fallows, G. Kaplan, Chronic pulmonary cavitary tuberculosis in rabbits: a failed host immune response. *Open Biol* **1**, 110016 (2011). Briefly, rabbits were exposed to *Mtb*-containing aerosol using a nose-only delivery system. Three hours post-infection, three rabbits were euthanized, and serial dilutions of the lung homogenates were cultured on Middlebrook 7H11 agar plates to enumerate the number of bacterial colony forming units (CFUs) implanted in the lungs. The infection was allowed to progress for 16 to 20 weeks. Two groups of two infected rabbits received doxycycline in the chow at 200 or 400 ppm for seven consecutive days. Blood samples were collected in EDTA-coated or lithium heparin tubes pre-dose and at serial time points covering a period of 2 to 6 h. Blood samples were inverted 8 to 10 times and kept on ice for no more than 10 min prior to

centrifugation at 2000-3000 *g* for 10 min to recover plasma and quantify doxycycline by LC/MS-MS. Rabbits were euthanized following the last blood collection and lung lesions were dissected prior to homogenization and drug quantitation by LC-MS/MS as described above.

Rv3406 catalytic assay using Bio-AMS as a substrate

All reactions were performed in triplicate in 500 µL at 25 ºC. The complete assay contained 1 mM Bio-AMS, 1 mM alpha-ketoglutarate (α-KG), and 3 µM Rv3406 in assay buffer (40 mM Tris acetate buffer, pH 7.5, 50 mM NaCl, 0.2% triton X-100, 100 µM iron (II) chloride, 2 mM ascorbate). Reactions were initiated by the addition of Rv3406. Negative control reactions lacked Rv3406. Aliquots (20 µL) were withdrawn at 0, 30, 60, 120, 240, 360, and 1440 min and immediately quenched with 20 µL 10% TCA (trichloroacetic acid) to precipitate the protein. Adenosine was used as an internal standard with a final concentration of 0.17 mM. Samples were then centrifuged for 7 min at 16,500 *g*, 30 µL supernatant was removed and stored at –80 ºC until further HPLC analysis. For the steady-state kinetic studies, incubations identical to those described above were set up. Seven different concentrations of Bio-AMS were used (4, 2, 1, 0.5, 0.25, 0.125, and 0.0625 mM), 20 µL aliquots were taken at 0 min and 60 min (initial velocity conditions), and protein was precipitated by the addition of 20 µL of 10% TCA solution, which included 0.1 µM 3′-deoxy-3′-fluoro-5′-*O*-[*N*-(salicyl)sulfamoyl]adenosine (1) as an internal standard. The remainder of the workup was identical to that described above. The concentration of metabolite **4** was quantified by LC-MS/MS to provide the initial velocity, v_0 at each concentration of Bio-AMS. The plot of v_0 versus [Bio-AMS] (Fig. 2c) was fit to the Michaelis-Menten equation to determine the kinetic parameters, k_{cat} and K_M.

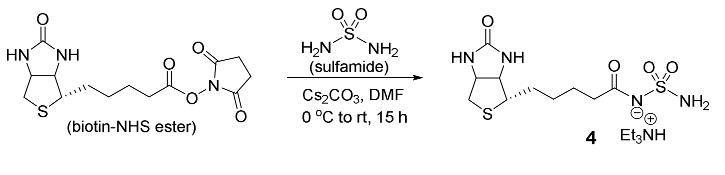

(1)

Compound **4** was synthesized in a single step by the reaction of biotin-NHS ester with commercially available sulfamide (eq 1). To a solution of sulfamide (0.11 g, 1.17 mmol, 4.0 equiv) and biotin-NHS ester (0.10 g, 0.29 mmol, 1.0 equiv) in DMF (10 mL) cooled to 0 ºC was added Cs_2CO_3 (0.29 g, 0.88 mmol, 3.0 equiv) and the reaction mixture was stirred at that temperature for 30 min. The reaction mixture was allowed to warm to room temperature and stirred for 15 h. The reaction mixture was filtered, solvent was evaporated, and re-evaporated with 5% MeOH in CH_2Cl_2 (×2) under reduced pressure. Purification of the residue by preparative reverse-phase HPLC using a Phenomenex Gemini 10 μm C18 (250 × 21.2 mm) column at a flow rate of 21 mL/min with a gradient from 5% to 50% MeCN in 20 mM aqueous triethylammonium bicarbonate (pH 7.5) over 10 min, followed by isocratic elution with 50% MeCN for more 5 min. The total run time was 15 min and the detection wavelength was 210 nm. The appropriate fractions containing the product were pooled and lyophilized to afford the product *N*-(biotinoyl)sulfamide **4** (70 mg, 56%) as white powder. ^1H NMR (400 MHz, [D_6]DMSO) δ 6.94 (br s, 2H), 6.41 (br s, 1H), 6.33 (br s, 1 H), 4.30 (dd, *J* = 7.8, 5.0 Hz, 1H), 4.13 (ddd, *J* = 7.5, 4.6, 1.9 Hz, 1H), 3.10 (ddd, *J* = 8.5, 6.2, 4.4 Hz, 1H), 2.82 (dd, *J* = 12.4, 5.0 Hz, 2H), 2.56 (q, *J* = 7.2 Hz, 6H Et_3N), 2.13 (t, *J* = 7.4 Hz, 2H), 1.66-1.44 (m, 4H), 1.36-1.27 (m, 2H), 0.98 (t, *J* = 7.2 Hz, 9H Et_3N); ^1H NMR (400 MHz, MeOD) δ 4.49 (dd, *J* = 7.9, 4.9 Hz, 1H), 4.32 (dd, *J* = 7.9, 4.4 Hz, 1H), 3.22 (ddd, *J* = 8.3, 6.1, 4.3 Hz, 1H), 3.03 (q, *J* = 7.3 Hz, 6H Et_3N), 2.93 (dd, *J* = 12.7, 5.0 Hz, 1H), 2.70 (d, *J* = 12.7 Hz, 1H), 2.13 (dd, *J* = 8.3, 6.8 Hz, 2H), 1.80-1.54 (m, 4H), 1.50-1.42 (m, 2H), 1.24 (t, *J* = 7.3 Hz, 9H Et_3N); ^{13}C NMR (100 MHz, [D_6]DMSO) δ 172.6, 162.7, 61.0, 59.2, 55.4, 45.7, 39.9, 35.7, 28.1, 28.0, 24.6, 10.8; HRMS (ESI–) *m*/*z* calculated for $C_{10}H_{17}N_4O_4S_2^-$ [$(M-H)^-$] 321.0697, found 321.0691 (Δ –1.9 ppm).

LC-MS/MS Analysis to identify Bio-AMS degradation products generated by Rv3406

Samples were analyzed by LC-MS/MS (Shimadzu UFLC XR-AB SCIEX QTRAP 5500). Reverse-phase LC was performed on a Kinetix C18 column (50 mm × 2.1 mm, 2.6 μm particle

size; Phenomenex, Torrance, CA). Mobile phase A was 0.1% aqueous formic acid while mobile phase B was 0.1% formic acid in acetonitrile. Initial conditions were 5% B from 0 to 0.5 min, after which the %B was increased to 95% from 0.5 to 3 min. The column was washed in 95% B for 2 min, returned to 5% over 0.2 min, and allowed to re-equilibrate for 2.8 min in 5% B to provide a total run time of 8 min. The flow rate was 0.5 mL/min and the column oven was maintained at 40 °C. The injection volume was 10 µL. All analytes were analyzed by MS in positive ionization mode by Multiple Reaction Monitoring (MRM). To determine the optimum MRM settings, the metabolite **4** was infused at a concentration of 10 µM (in 1:1 water/acetonitrile containing 0.1% formic acid) onto the MS by a syringe pump at a flow of 10 µL/min. For compound **4**, precursor ion was detected at m/z 323, fragment ion at 227.2 with a collision energy of 25 V and declustering potential of 35 V. Analyte and internal standard peak areas were calculated (MultiQuant, version 2.0.2, AB SCIEX). Analyte peak areas were normalized to internal standard peak areas and the analyte concentrations were determined with an appropriate standard curve.

HPLC Analysis of Bio-AMS and other metabolites for intrabacterial metabolic analyses

Samples were analyzed by reverse phase HPLC (Agilent 1260) on a Phenomenex Gemini C18 column (5 micron, 4.6 x 250 mm) with a flow rate of 1 mL/min. Mobile phase A was 20 mM TEAB (triethylammonium bicarbonate) buffer pH 7.3 and mobile phase B was acetonitrile. Initial conditions were 2% B and the %B was increased to 20% over 10 min, and to 95% from 10 to 13 min. The column was washed with 95% B for 3 min, returned to 5% over 1 min, and allowed to re-equilibrate for 4 min in 5% B to provide a total run time of 21 min. The injection volume was 20 µL. The UV detection was carried out at 240 nm and peaks were collected manually to be analyzed by mass spectrometry.

Analyses of Bio-AMS and metabolite 4 in *Mtb*

A previously described (J. H. Kim, K. M. O'Brien, R. Sharma, H. I. Boshoff, G. Rehren, S. Chakraborty, J. B. Wallach, M. Monteleone, D. J. Wilson, C. C. Aldrich, C. E. Barry, 3rd, K. Y. Rhee, S. Ehrt, D. Schnappinger, A genetic strategy to identify targets for the development of drugs that prevent bacterial persistence. *Proc Natl Acad Sci U S A* **110**, 19095-19100 (2013) experimental set up with some modifications was used to study accumulation of Bio-AMS and its degradation products inside *Mtb*. Briefly, *Mtb* laden filters were grown in Middlebrook 7H10 agar plates for 5 days followed by exposure to Bio-AMS for 18 h in GAST medium containing 25 µM Bio-AMS or an equivalent amount of DMSO. After 18 h the filters were incubated on GAST medium for 24 h without any antibiotics and samples were collected and processed as described.

Quantitation of Bio-AMS and biotin in mouse plasma

Bio-AMS and its metabolites *N*-(biotinoyl)sulfamide **4** and biotin were extracted by diluting 20 µL of mouse plasma with 20 µL of acetonitrile:water (1:1), and 180 µL of methanol:acetonitrile (1:1) containing 10 ng/mL of verapamil (Toronto Research Chemicals, Inc) as an internal standard (IS). Due to the endogenous presence of biotin in plasma, a biotin-d4 (Toronto Research Chemicals, Inc) standard curve was used to quantify the biotin concentration. To precipitate the proteins, the mixture was vortexed for 5 min, centrifuged at 3000 *g* for 5 min, and 100 µL of the supernatant was recovered and mixed with 100 µL of Milli-Q water for mass spectrometric analysis. LC/MS analysis was performed with an Agilent 1260 liquid chromatography system coupled to a 4000 Qtrap mass spectrometer (AB Sciex) in MRM (multiple reaction monitoring) mode with positive electrospray ionization (ESI), and an Agilent column SB-C8, 4.6 $\times$ 75mm, 3.5 µm. Mobile phase A was 0.1% formic acid in 100% H_2O and mobile phase B was 0.1% formic acid in 100% acetonitrile. Injection volumes were routinely 10 µL. Bio-AMS and the two metabolites **4**, and biotin were quantified. The ions monitored were Bio-AMS (*m/z*

572.02/136.20), **4** (346.10/136.00), biotin (245.1/97.1), biotin-d4 (249.10/97.10), and verapamil (*m/z* 455.40/165.20) for quantification by constructing a linear calibration using the Analyte/IS peak area ratios. The lower limits of quantification (LLOQ) for Bio-AMS, **4**, and biotin respectively were 10, 5, and 5 ng/mL, and the higher limits of quantification (HLOQ) were 20, 10, and 5 μg/mL.

SUPPLEMENTARY FIGURES

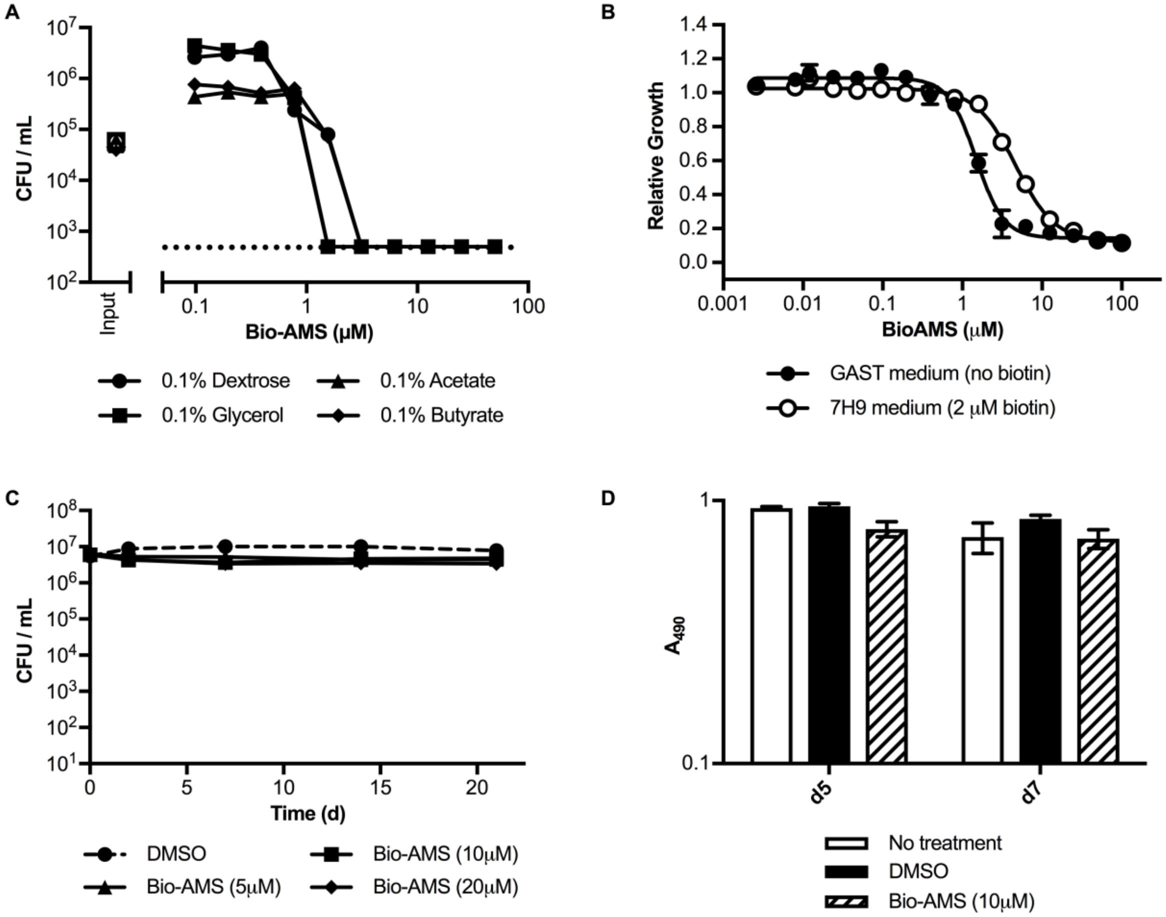

Fig. S1. Bio-AMS kills *Mtb* in medium with different carbon sources and is not acutely toxic to mouse macrophages.

A. Minimal bactericidal concentration of Bio-AMS against *Mtb* H37Rv strain in media with different carbon sources. The minimal bactericidal concentration of Bio-AMS was determined by culturing serial dilutions on 7H11 agar medium for CFUs on d14 and counting

the recovered CFUs after 3 weeks of incubation. The limit of detection was 500 CFUs and is indicated by the dotted line. Data are representative of at least two independent experiments.

B. Impact of Bio-AMS on growth of *Mtb* H37Rv strain in liquid media with and without biotin.

C. Impact of Bio-AMS on viability of non-replicating *Mtb* H37Rv strain. As in (A) except that *Mtb* was starved in PBS Tyloxapol (PBS-T) for 10 days prior to addition of compound on d0 and kept in PBS-T before aliquots were plated to determine CFU.

D. Toxicity of Bio-AMS to mouse macrophages. Viability was assessed using an MTS assay. Data are averages of 3 cultures ± SEM.

Compound	Top Conc. Tested (µM)	Glucose IC50 (µM)	Galactose IC50 (µM)	Glu/Gal Fold Change	Comments
Chlorpromazine	100	10.74	8.927	1.20	negative control
Papaverine	100	13.18	1.271	10.37	positive control
Rotenone	25	0.2554	<0.01	>25	positive control
Bio-AMS	100	56.87	53.91	1.05	test compound

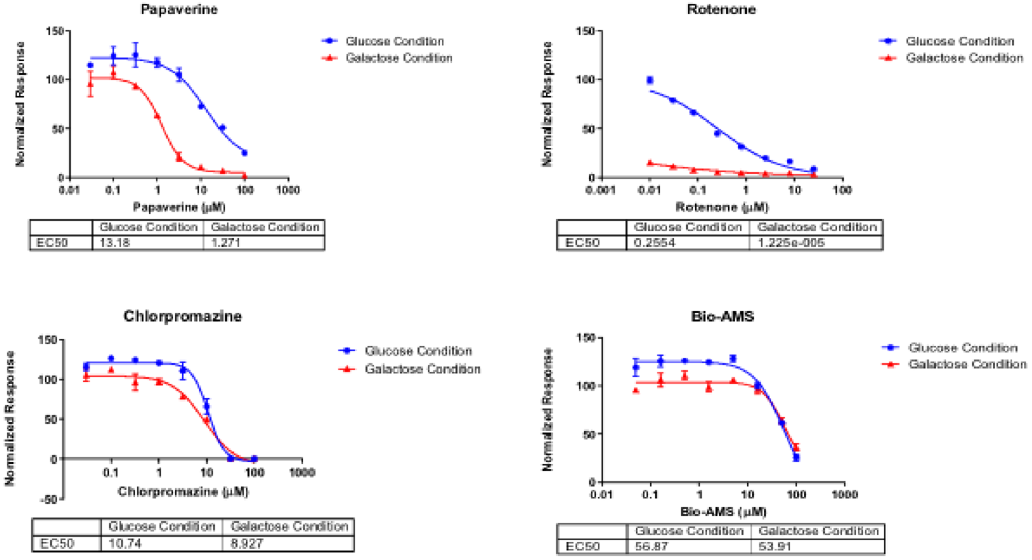

Fig. S2. Evaluation of mitochondrial toxicity.

Cytotoxicity was measured with HepG2 cells grown in glucose and galactose media. Under galactose media, cells are more susceptible to mitochondrial toxicity. Mitochondrial toxicity was determined by calculating the fold change in IC50 value observed in the glucose medium compared to the galactose medium. Rotenone and papaverine were included as positive controls for mitochondrial toxicity; chlorpromazine served as the negative control. A 3-fold or larger shift in the IC50 is indicative of mitochondrial toxicity. The shift observed for Bio-AMS was 1.05-fold. Data were provided by Cyprotex.

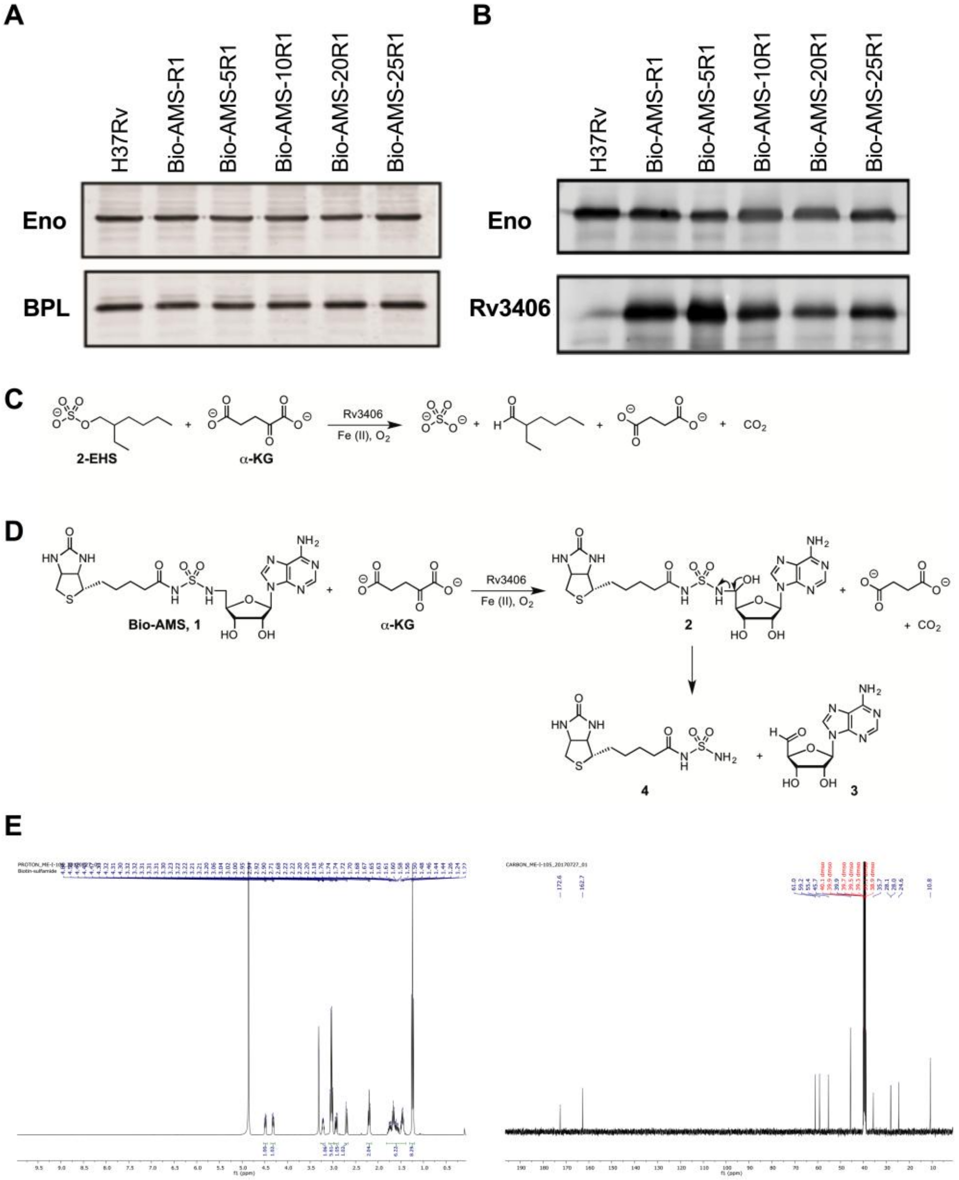

Fig. S3. Emergence of *Mtb* mutants resistant to Bio-AMS.

A. Expression of BPL. Immunoblots of total protein extracts form *Mtb* H37Rv strain and Bio-AMS resistant isolates with antisera against BPL and enolase (eno). Eno was used as the loading control.

B. Expression of Rv3406. Immunoblots of total protein extracts form *Mtb* H37Rv strain and Bio-AMS resistant isolates with antisera against Rv3406 and eno.

C. Biochemical reaction catalyzed by Rv3406.

D. Proposed reaction of Rv3406 with Bio-AMS

E. Characterization of compound 4 by NMR. 44 MHz ^{1}H NMR of compound 4 in MeOD (upper panel) and [D$_6$]DMSO.

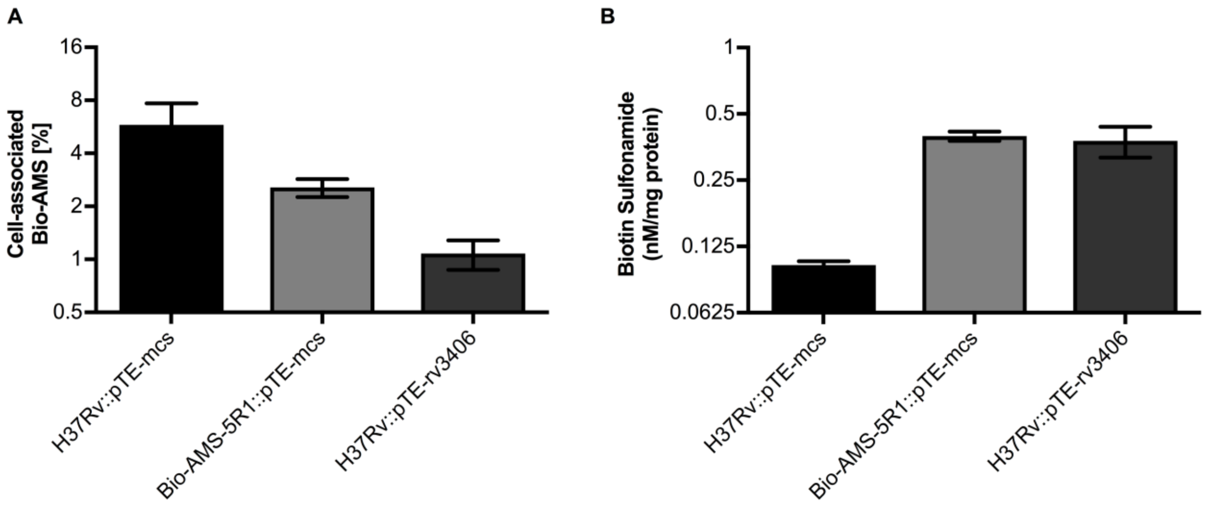

Fig. S4. Quantification of *Mtb*-associated Bio-AMS and biotin sulfonamide.

A. Relative amount of Bio-AMS associated with *Mtb* H37Rv strain. Bio-AMS was measured after exposing *Mtb* to Bio-AMS in vitro for 18 hours followed by 24 hours of incubation in Bio-AMS-free medium. The bars represent the amount of Bio-AMS remaining in Mtb after 24 hours of incubation in drug free medium compared to the total Bio-AMS accumulated after 18h of drug exposure.

B. Amount of cell-associated biotin sulfonamide.

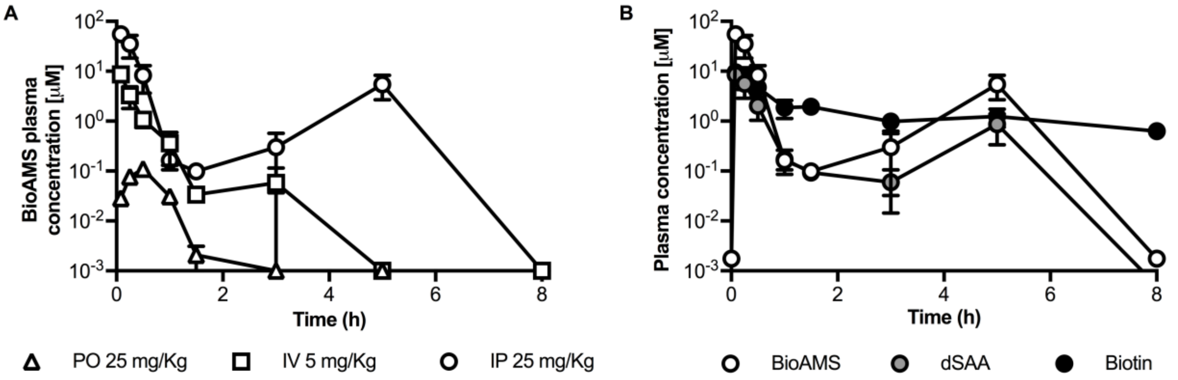

Fig. S5. PK profiles and metabolism of Bio-AMS in mice.

Bio-AMS was rapidly eliminated following IV and IP administration. An apparent rebound was observed in the IP arm at 5 h post dose, possibly associated with enterohepatic cycling. IP administration provided good exposure but exposure following oral gavage was low, with a bioavailability of 2%. Since Bio-AMS is highly soluble, poor permeability and/or first pass metabolism could be responsible for the very low oral bioavailability.

A. Pharmacokinetic concentration-time profiles. The pharmacokinetic profiles of Bio-AMS in mice were determined following intravenous (IV), intraperitoneal (IP) and oral (PO) administration. Samples were collected at time points indicated. Average plasma concentrations are shown with standard deviations (n=4).

B. *In vivo* quantitative metabolism of Bio-AMS in mice following intraperitoneal injection of a single 25 mg/kg dose. Endogenous biotin concentrations in plasma (~50 ng/mL) were subtracted from the measured biotin concentrations. Average plasma concentrations are shown with standard deviations (n=4).

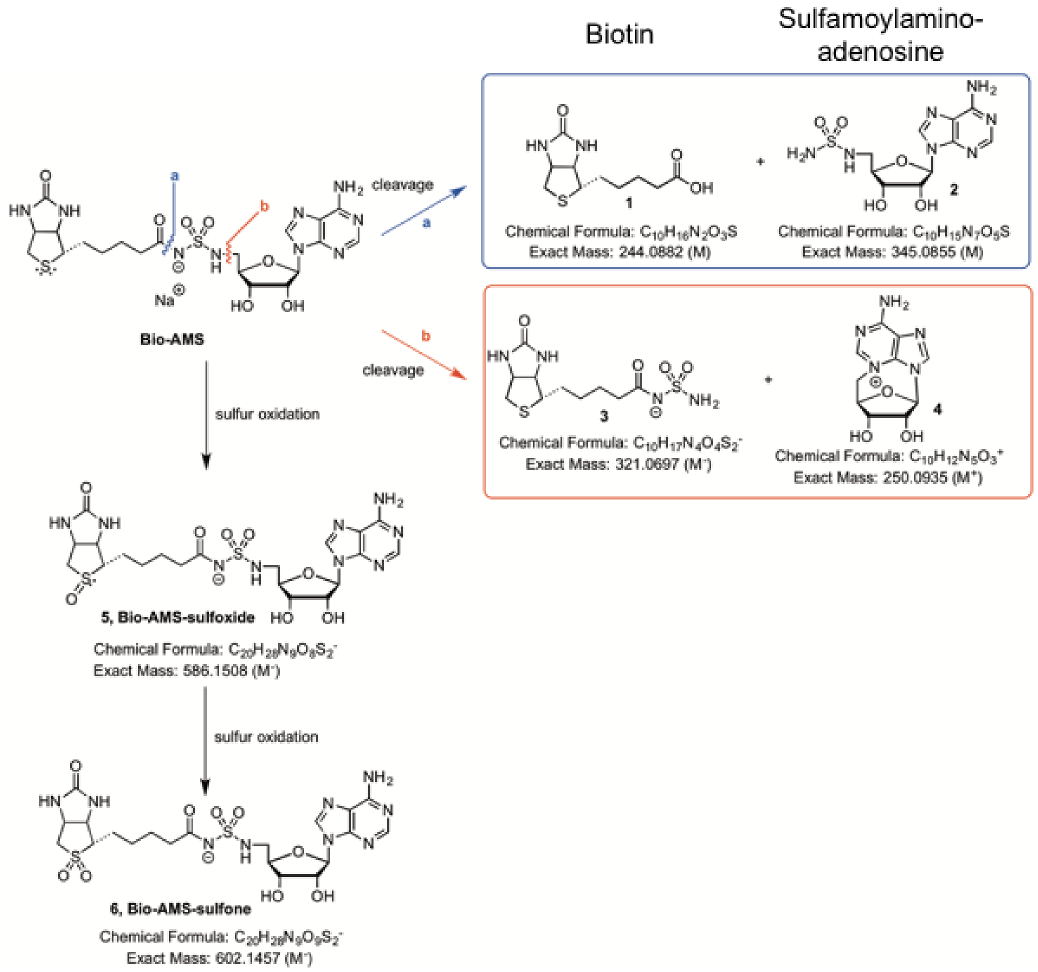

Fig. S6. Putative Bio-AMS metabolic and degradation pathways.

Biotin and sulfamoylamino-adenosine were detected as the major metabolites *in vivo*.

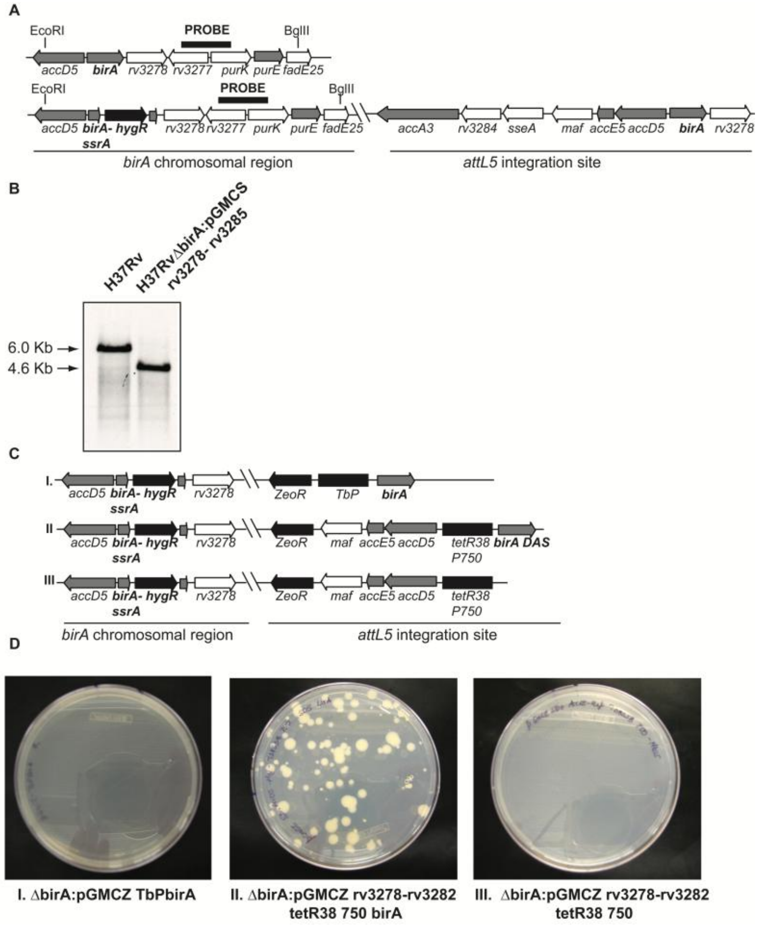

Fig. S7. Construction of the *Mtb* BPL-DUC strain.

We previously reported *M. smegmatis birA* mutants, which were constructed using a strain that contained a second copy of *birA* integrated into the attachment site of the phage L5 (attL5) (B. P. Duckworth, T. W. Geders, D. Tiwari, H. I. Boshoff, P. A. Sibbald, C. E. Barry, 3rd, D. Schnappinger, B. C. Finzel, C. C. Aldrich, Bisubstrate adenylation inhibitors of biotin protein ligase from *Mycobacterium tuberculosis*. *Chem Biol* **18**, 1432-1441 (2011). To apply this strategy to *Mtb* H37Rv strain, we integrated a second copy of *birA* into the attL5 of *Mtb* and then attempted to delete 384 bp from the middle of *birA*. We left the 5' and 3' ends of *birA* intact because we expected that this would avoid polar effects on the gene upstream and downstream of *birA*. We furthermore designed the KO cassette so that recombination added the *ssrA*-tag to the remaining 5'-part of *birA* ensure degradation of the truncated BPL by the Clp proteases. However, all our attempts to obtain *birA* mutants in *Mtb* using this strategy were unsuccessful. We hypothesized this was because we did not achieve our goal of avoiding a polar effect on essential genes located upstream of *birA*. We therefore generated an *Mtb* strain, attL5::*birA-accA3*, that contained not only *birA* but also its upstream genes in attL5. This strain permitted isolation of a mutant, Δ*birA* attL5::*birA-accA3*, in which the native copy of *birA* was inactivated by allelic exchange. By reducing the number of *birA* upstream genes in the attL5 we then

demonstrated that *Mtb* Δ*birA* was only viable when additional copies of *accD5*, *accE5*, and *maf*, but not *sseA*, *rv3284*, and *accA3*, where provided. We therefore used *Mtb* Δ*birA* attL5::*birA*-*accE5* to construct *Mtb* BPL-DUC, which constitutively expressed *accD5*, *accE5*, and *maf* but permitted to deplete BPL via a dual-control switch (D. Schnappinger, K. M. O'Brien, S. Ehrt, Construction of conditional knockdown mutants in mycobacteria. *Methods Mol Biol* 1285, 151-175 (2015)) that combines transcriptional silencing with controlled proteolysis.

A. Schematic showing the genomic region of *birA* in H37Rv and H37Rv::Δ*birA* that expresses the genes in birA region from *rv3278*- *rv3285* at the attL5 locus. The *EcoR*I and *Bgl*II restriction sites and the location of probe used for southern blotting are indicated.

B. Southern blot using genomic DNA from H37Rv and H37Rv::Δ*birA* digested with *Eco*RI and *Bgl*II and probed with the DNA fragment indicated in (a).

C. Schematic of constructs used to perform allelic exchanges in *birA* mutant to establish essentiality of *birA*.

D. Replacement transformation performed using the constructs described in (c) that swap the antibiotic resistance of the strains from streptomycin to zeocin if the allelic exchange is viable for bacteria. The transformations were plated on 7H10 agar plates containing zeocin (25 µg/ml) and were incubated for 4 weeks. Colonies obtained (if any) were checked for streptomycin sensitivity to rule out co-integration.

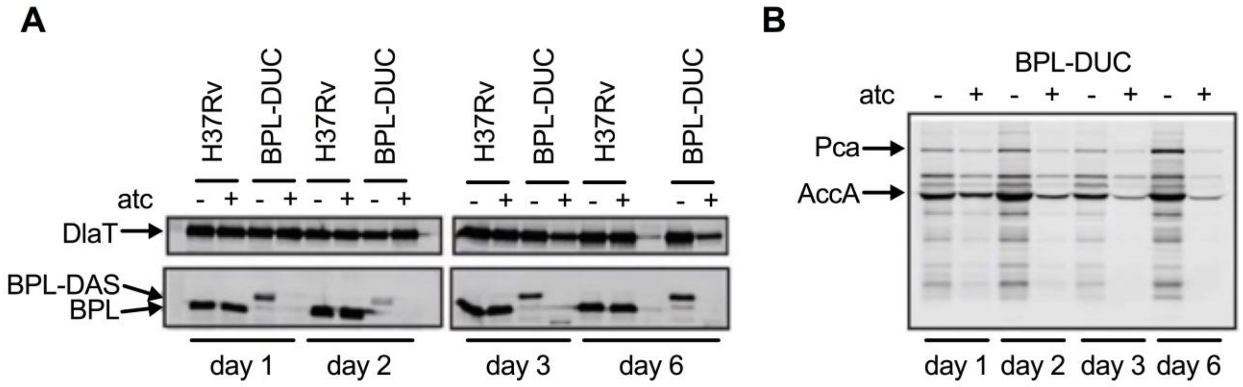

Fig. S8. Impact of atc on BPL expression and protein biotinylation.

A. Immunoblot for BPL. 20 ug of total protein extract were resolved on 10% SDS PAGE gels and analyzed with BPL-specific antisera. Antiserum against DlaT was used to confirm equal loading.

B. Immunoblot for biotinylated proteins. As (B) except that streptavidin was used instead of the BPL-specific antiserum.

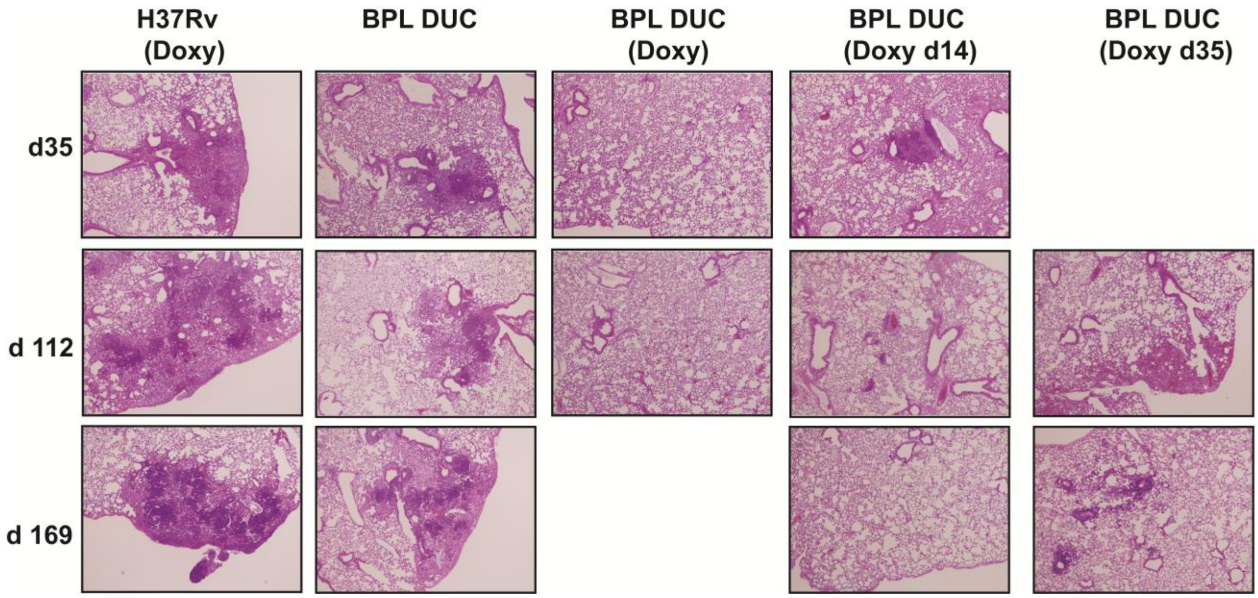

Fig. S9. Histopathology of lungs infected with the *Mtb* BPL-DUC strain.

Left lobes of the recovered lungs were fixed in 10% formalin. Gross pathology was assessed and H&E staining was performed for lung sections of mice infected with BPL-DUC.

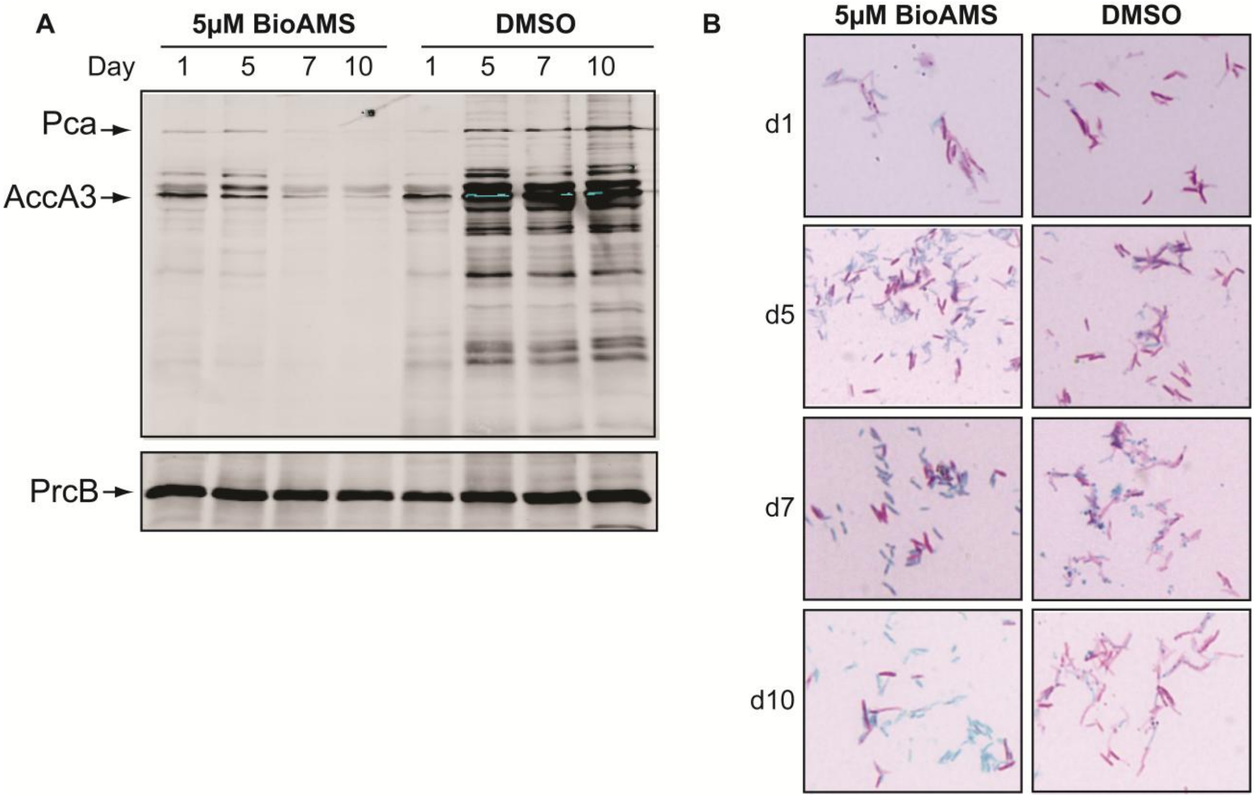

Fig. S10. Bio-AMS treatment inhibits protein biotinylation and results in loss of *Mtb* acid-fastness.

A. Immunoblot for biotinylated proteins. Actively growing *Mtb* culture was exposed to DMSO or 5 µM Bio-AMS for the indicated duration. At each time point, aliquots of culture were removed for immunoblotting with IRdye800 CW streptavidin antibody to detect protein biotinylation. PrcB was used as the loading control.

B. Acid fastness. Bacteria were grown as for (a) and Kinyoun staining on formalin-fixed bacteria was used to assess acid-fastness.

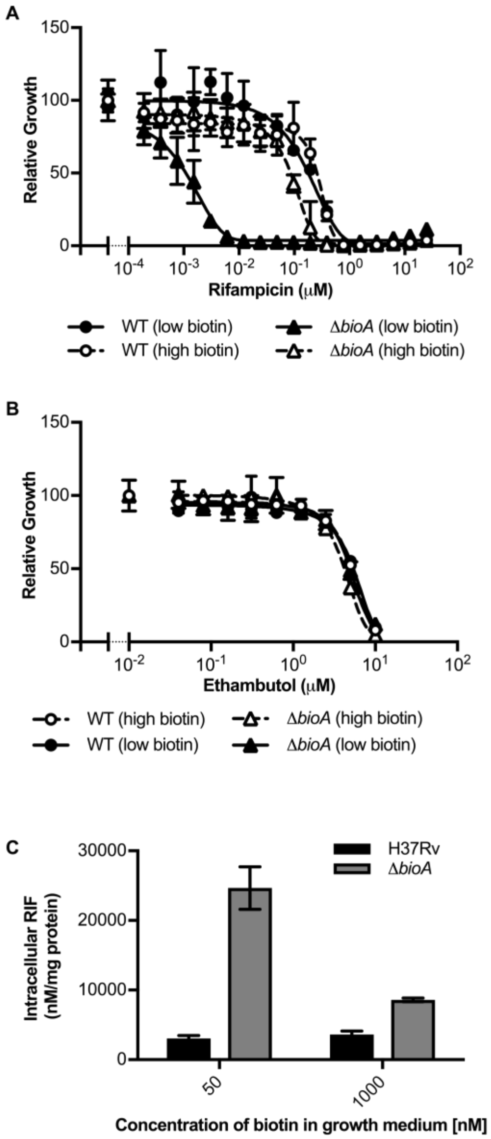

Fig. S11. Growth of *Mtb* Δ*bioA* in low concentrations of biotin increases potency of rifampicin but not ethambutol.

A. Wildtype and Δ*bioA* were grown to mid log phase and susceptibility to rifampicin were determined in Sauton`s medium containing low (50 nM) or high (1000 nM) biotin. Growth was assessed by measuring the optical density after 14 days of exposure to the compounds and displayed as percentage growth compared to respective no drug control.

B. Same as (a) but with ethambutol instead of rifampicin.

C. Wildtype and *Mtb* Δ*bioA* were grown with low (50 nM) or high (1000 nM) biotin and intracellular rifampicin was quantified by LC/MS.

SUPPLEMENTARY TABLES

Table S1. Whole-genome sequencing of Bio-AMS–resistant *Mtb* strains.

Strain	Bio-AMS[a]	Mutations
Bio-AMS R1	10 µM	***rv3405c*: -C aa 16**; ppsA:+G aa 1467; lhr: D638G
Bio-AMS 5R1	25 µM	***rv3405c*: -G in aa 77**; IS6110::Rv1358
Bio-AMS 10R1	50 µM	***rv3405c*: Q25***; IS6110::Rv1358
Bio-AMS 20R1	100 µM	***rv3405c*: P36R**; mce3C:I241L; ppsD:E1490*; IS6110::Rv1358
Bio-AMS 25R1	125 µM	***rv3405c*: S40***; IS6110::Rv1358

[a] Concentration used for strain isolation

* Mutated to a stop codon.

Table S2. Genes whose transcripts changed more than threefold in three Bio-AMS–resistant strains.

Gene	mRNA abundance (mutant / H37Rv)		
	Bio-AMS 5R1	Bio-AMS 10R1	Bio-AMS 25R1
rv3406	232.7	190.4	202
rv3405c	14.8	11.5	10.7
pe13	4.8	13.3	16.2
rv3407	3.6	3	3
rv2989	3.5	5.7	7.5
leuC	3.3	8.1	9.9
ppsB	-3.6	-3.7	-3.1
ppsC	-4.4	-4	-4.1
ppsA	-4.8	-3.1	-3.2

Table S3. Kinetic parameters of *Mtb* Rv3406.[a]

Substrate	k_{cat} (min^{-1})	K_M (mM)	k_{cat}/K_M (min^{-1} mM^{-1})
α-KG[b]	21.8 ± 1.0	0.0094 ± 0.0012	2319 ± 106
2-EHS[b]	19.2 ± 0.9	0.0088 ± 0.0005	2186 ± 97
Bio-AMS 1	0.015 ± 0.001	0.424 ± 0.136	0.035 ± 0.007

[a] Initial velocities of the Rv3406 reaction were assayed by direct detection of products using LC-MS/MS as described in the text. All assays were performed at 25 °C and pH 7.5.

[b] J. Neres, R. C. Hartkoorn, L. R. Chiarelli, R. Gadupudi, M. R. Pasca, G. Mori, A. Venturelli, S. Savina, V. Makarov, G. S. Kolly, E. Molteni, C. Binda, N. Dhar, S. Ferrari, P. Brodin, V. Delorme, V. Landry, A. L. de Jesus Lopes Ribeiro, D. Farina, P. Saxena, F. Pojer, A. Carta, R. Luciani, A. Porta, G. Zanoni, E. De Rossi, M. P. Costi, G. Riccardi, S. T. Cole, 2-Carboxyquinoxalines kill mycobacterium tuberculosis through noncovalent inhibition of DprE1. *ACS Chem Biol* **10**, 705-714 (2015).

Table S4. PK parameters of Bio-AMS after intravenous, intraperitoneal, and oral administration.[a]

**Route of administration**	**Dose** (mg/kg)	**$AUC_{[0-8]}$[b] (μM*h)** Average (SD)	**C_{max}[c] (μM)** Average (SD)	**F[d]** (%)
IV	5	2.50 (1.08)	8.72 (2.29)	n/a
PO	25	0.26 (0.08)	0.11 (0.03)	2
IP	25	31.77 (10.95)	61.10 (15.39)	241 [e]
IP – SAA released	NA	5.55	8.57	n/a
IP – biotin released	NA	13.66	9.51	n/a

[a] CD-1 outbred mice are the most common multipurpose rodent model for pharmacokinetics and tolerability studies. Parameters for the two major metabolites measured in mice dosed IP are included.

[b] $AUC_{[0-8]}$: area under the concentration-time curve from 0 to 8h post dose;

[c] C_{max}: peak plasma concentration;

[d] F: bioavailability relative to IV exposure.

[e] Apparent F greater than 100% possibly due to (1) first IV timepoint not close enough to dosing time (5 min) thus underestimating IV exposure and (2) enterohepatic cycling seen following IP dosing

Table S5. Tolerability of Bio-AMS at ascending intraperitoneal doses in CD-1 mice.[a]

Bio-AMS 50mg/kg (IP) 4 ml/kg

Mouse #	Weight	½ h Observation	24h Observation
1	28	Active, grooming	Normal activity
2	24	Active, grooming	Scruffy; some lethargic
3	28	Active, grooming	Normal activity

Bio-AMS 100mg/kg (IP) 4 ml/kg

Mouse #	Weight	½ h Observation	24h Observation
4	35	Normal activity	Normal activity; grooming
5	32	Normal activity	Dead
6	29	Normal activity	Euthanized

Bio-AMS 250mg/kg (IP) 4 ml/kg

Mouse #	Weight	½ h Observation	24h Observation
7	28	Heavy breathing	Dead
8	25	Scruffy, heavy breathing	Dead
9	32	Normal activity	Euthanized

Bio-AMS 500mg/kg (IP) 4 ml/kg

Mouse #	Weight	½ h Observation	24h Observation
10	37	Scruffy, heavy breathing	Near death; scruffy; pale color
11	27	Scruffy, heavy breathing	Euthanized
12	29	Scruffy, heavy breathing	Euthanized

[a] These data were generated to determine whether the projected efficacious dose of Bio-AMS would be tolerated in mice (with plasma concentrations above the MIC for a minimum of 40-50% of the dosing interval). For this, we conducted a dose escalation tolerability study in which groups of three mice received ascending IP doses of Bio-AMS. Only the lowest dose of 50 mg/kg was tolerated up to 24 h post injection. Animal weights remained unchanged during the 24 h period post-injection at all doses. Assuming linear pharmacokinetics, a 50 mg/kg dose would deliver plasma concentrations above the MIC for a very small fraction of the dosing interval, if at all.

Table S6. Concentrations of rifampicin and doxycycline in the plasma of CD-1 mice receiving rifampicin alone or rifampicin with doxycycline in the diet after a single dose (10 mg/kg) of rifampicin and at a steady state.

Parameter [b]	Rifampicin single dose	Rifampicin single dose plus doxycycline in diet [a]	Rifampicin steady state	Rifampicin steady state + doxycycline in diet [*]
	Mean (SD); n = 4			
Rifampicin C_{max} (μg/mL)	5.21 (1.45)	6.23 (1.93)	6.68 (1.70)	5.49 (2.14)
Rifampicin $AUC_{[0-8]}$ (μg*h/mL)	30.74 (8.53)	32.14 (4.60)	39.62 (9.88)	30.98 (10.55)
Doxycycline C_{max} (μg/mL) [c]	n/a	0.83 (0.26)	n/a	0.73 (0.09)
Doxycycline C_{min} (μg/mL) [c]	n/a	0.36 (0.09)	n/a	0.26 (0.02)
Doxycycline $AUC_{[0-8]}$ (μg*h/mL)	n/a	4.78 (0.91) [d]	n/a	3.36 (0.97) [d]

[a] Doxy was supplied in food pellets at 2000 ppm for 7 days

[b] Doses and dosing schedules are described in Methods. Steady state: 7 daily rifampicin doses; AUC: Area Under the concentration-time Curve; C_{max}: peak plasma concentration; C_{min}: trough plasma concentration

[c] Doxy concentrations were measured during daytime while mice are nocturnal and eat more at night, thus DOXY PK parameters are conservative estimates at the lower boundary of the range achieved over the course of a given 24h period

[d] Difference between doxycycline AUC with a single rifampicin dose versus 7 rifampicin doses statistically significant: *P* value = 0.0244 (unpaired parametric *t*-test assuming unequal standard deviations)

Table S7. Distribution of doxycycline in *Mtb*-infected rabbit lung lesions relative to plasma after administration of doxycycline in chow for 7 days.

Animal ID	1066	1069	1070	1071
Doxy concentration (ppm in chow)	200	200	400	400
Average lesion/plasma ratio (SD)	6.1 (4.3)	3.9 (2.6)	3.7 (2.5)	5.4 (2.5)
N (number of lesions)	30	44	10	19

[a] Lesion distribution studies were conducted in rabbits, which generate granulomas that are sufficiently large for individual dissection and processing. Mouse granulomas are poorly organized and not large enough to enable drug quantitation at the lesion level.

Table S8. Strains and plasmids.

Strains and plasmids	Description	Source
M. tuberculosis H37Rv	*Mycobacterium tuberculosis* H37Rv	S. Gandotra, D. Schnappinger, M. Monteleone, W. Hillen, S. Ehrt, In vivo gene silencing identifies the Mycobacterium tuberculosis proteasome as essential for the bacteria to persist in mice. *Nat Med* **13**, 1515-1520 (2007).
H37Rv::pGMEH-PTb38-*rv3406*	Hyg^R; H37Rv strain overexpressing *rv3406* under *PTb38* promoter	This study
H37Rv::pTE-mcs	Hyg^R; H37Rv strain	This study
BioAMS5R1::pTE-mcs	Hyg^R; BioAMS resistant strain	This study
BPL-DUC	Hyg^R Kan^R Zeo^R; *ΔbirA:pGMCZ accD5-maf tetR38 750 SD5 birA DAS:pGMCtK TSC10M1 sspB*	This study
bioA tetON-1	Hyg^R; *bioA* overexpressor with tet regulated expression of *bioA*	S. Woong Park, M. Klotzsche, D. J. Wilson, H. I. Boshoff, H. Eoh, U. Manjunatha, A. Blumenthal, K. Rhee, C. E. Barry, 3rd, C. C. Aldrich, S. Ehrt, D. Schnappinger, Evaluating the sensitivity of Mycobacterium tuberculosis to biotin deprivation using regulated gene expression. *PLoS Pathog* **7**, e1002264 (2011).
ΔbioA	Hyg^R; *bioA* knockout	S. Woong Park, M. Klotzsche, D. J. Wilson, H. I. Boshoff, H. Eoh, U. Manjunatha, A. Blumenthal, K. Rhee, C. E. Barry, 3rd, C. C. Aldrich, S. Ehrt, D. Schnappinger, Evaluating the sensitivity of Mycobacterium tuberculosis to biotin deprivation using regulated gene expression. *PLoS Pathog* **7**, e1002264 (2011).
H37Rv::pGMCS-*rv3278-rv3284*	$Strep^R$; used for mutant construction; expresses all the genes from *rv3278-rv3284* at *attL5* site.	This study
ΔbirA::pGMCS-*rv3278-rv3284*	Hyg^R $Strep^R$; *birA* mutant with 400 bp of the gene replaced by Hyg resistant cassette expressing all	This study

	the genes from *rv3278-rv3284* at *attL5* site.	
Δ*birA*:pGMCZ-*rv3278-rv3282-tetR38*-750-*birA*	Hyg^R Zeo^R; constitutively expresses *rv3278-rv3282* and *birA* under tet regulated promoter at *attL5* site	This study
pGMEH-PTb38-*rv3406*	Hyg^R; *rv3406* gene cloned under *PTb38* promoter; replicates episomally.	This study
pGMCS-rv3278-*rv3284*	$Strep^R$; integrates to *attL5* site; contains *rv3278* to *rv3284*.	This study
pGMCZ-TbP*birA*	Zeo^R; *birA* cloned under native promoter; integrates to *attL5* site.	This study
pGMCZ-*rv3278-rv3282-tetR38*-750-*birA*	Zeo^R; contains *rv3278* to *rv3282* cloned under native promoter and *birA* cloned under *PtetO4C5G.*	This study
pGMCZ-*rv3278-rv3282-tetR38*-750	Zeo^R; same as the plasmid above but lacks *birA*.	This study
pGMCZ-*accD5-maf-tetR38*-750-SD5-*birA*-DAS	Zeo^R; contains *accD5*, *accE5* and *maf* cloned under native promoter and DAS tagged *birA* under P750; integrates at *attL5* site.	This study
pGMCtK-TSC10M1-*sspB*	Kan^R; integrates at *att-tweety* site and expresses *sspB* hydrolase under *PtetO* and *tsc10.*	J. H. Kim, K. M. O'Brien, R. Sharma, H. I. Boshoff, G. Rehren, S. Chakraborty, J. B. Wallach, M. Monteleone, D. J. Wilson, C. C. Aldrich, C. E. Barry, 3rd, K. Y. Rhee, S. Ehrt, D. Schnappinger, A genetic strategy to identify targets for the development of drugs that prevent bacterial persistence. *Proc Natl Acad Sci U S A* **110**, 19095-19100 (2013).
